# Nucleophosmin Phosphorylation by v-Cyclin-CDK6 Controls KSHV Latency

**DOI:** 10.1371/journal.ppat.1000818

**Published:** 2010-03-19

**Authors:** Grzegorz Sarek, Annika Järviluoma, Henna M. Moore, Sari Tojkander, Salla Vartia, Peter Biberfeld, Marikki Laiho, Päivi M. Ojala

**Affiliations:** 1 Genome-Scale Biology Program, Biomedicum Helsinki & Institute of Biomedicine, University of Helsinki, Helsinki, Finland; 2 Molecular Cancer Biology Program, Biomedicum Helsinki & Haartman Institute, University of Helsinki, Helsinki, Finland; 3 Department of Pathology and Oncology, Karolinska Institute/Hospital, Stockholm, Sweden; 4 Department of Radiation Oncology and Molecular Radiation Sciences, The Johns Hopkins University School of Medicine, Baltimore, Maryland, United States of America; 5 Foundation for the Finnish Cancer Institute, Helsinki, Finland; Oregon Health & Science University, United States of America

## Abstract

Nucleophosmin (NPM) is a multifunctional nuclear phosphoprotein and a histone chaperone implicated in chromatin organization and transcription control. Oncogenic Kaposi's sarcoma herpesvirus (KSHV) is the etiological agent of Kaposi's sarcoma, primary effusion lymphoma (PEL) and multicentric Castleman disease (MCD). In the infected host cell KSHV displays two modes of infection, the latency and productive viral replication phases, involving extensive viral DNA replication and gene expression. A sustained balance between latency and reactivation to the productive infection state is essential for viral persistence and KSHV pathogenesis. Our study demonstrates that the KSHV v-cyclin and cellular CDK6 kinase phosphorylate NPM on threonine 199 (Thr199) in de novo and naturally KSHV-infected cells and that NPM is phosphorylated to the same site in primary KS tumors. Furthermore, v-cyclin-mediated phosphorylation of NPM engages the interaction between NPM and the latency-associated nuclear antigen LANA, a KSHV-encoded repressor of viral lytic replication. Strikingly, depletion of NPM in PEL cells leads to viral reactivation, and production of new infectious virus particles. Moreover, the phosphorylation of NPM negatively correlates with the level of spontaneous viral reactivation in PEL cells. This work demonstrates that NPM is a critical regulator of KSHV latency via functional interactions with v-cyclin and LANA.

## Author Summary

Latency is the predominant mode of viral persistence in KS and PEL tumors, and has a fundamental impact on KSHV tumorigenesis. Establishment and maintenance of latency involves a number of viral and cellular factors. This study provides a novel functional link between LANA and v-cyclin by showing that phosphorylation of nucleophosmin (NPM) by the v-cyclin-CDK6 kinase complex supports its interaction with LANA, and thus enables the transcriptional silencing of KSHV lytic genes needed for latency. These findings indicate that KSHV has evolved mechanisms to utilize host proteins for maintaining the latency, and underscores the role of NPM as a regulator of not only mammalian transcription but also of viral transcription. Taken together, our data suggests that a cellular protein, NPM, is a critical factor for the latency of this oncogenic human virus, and may thus represent an attractive novel target for intervention.

## Introduction

Nucleophosmin (NPM) is a multifunctional nucleolar phosphoprotein that constantly shuttles between nucleus and cytoplasm [1]. It functions as a molecular chaperone and has been linked to a number of cellular processes from ribosome maturation and transcriptional control to apoptosis (reviewed in [2]). NPM is reported to associate with unduplicated centrosomes and to dissociate from centrosomes upon phosphorylation on Thr199 by cyclin E (NM001238) -CDK2 (NM001798), which coincides with the initiation of DNA replication and centrosome duplication [3],[4]. It is often highly expressed in tumors, and the *NPM1* (M26697) gene is frequently targeted by genetic alterations in various lymphomas and leukemias (reviewed in [2]). NPM can contribute to oncogenesis through various mechanisms, and has been linked to both tumor promoting and suppressing processes.

Kaposi's sarcoma-associated herpesvirus (KSHV) is an oncogenic human DNA virus in the family of γ-herpesviruses. KSHV infection is associated with Kaposi's sarcoma (KS) and certain B-cell malignancies such as an AIDS-related form of non-Hodgkin lymphoma, called primary effusion lymphoma (PEL), and Multicentric Castleman's disease (reviewed in [5]). Similar to other herpesviruses, KSHV life cycle displays latent and lytic phases. Majority of the tumor cells in KS and PELs are latently infected [6]–[8]. Lytic replication phase can be induced (viral reactivation) by a variety of intra- and extracellular factors, including hypoxia, cytokines and chemical agents, such as histone deacetylase (HDAC) inhibitors or protein kinase C agonists (reviewed in [9]). In addition, interactions of the viral machinery with components of the cellular signaling pathways and cellular transcription factors play an important role in the viral reactivation.

Latency-associated nuclear antigen (LANA), the product of ORF73 gene (AF305694) tethers the circular viral DNA to the host chromosomes [10],[11], and is required for the maintenance of the KSHV episomal genome [12],[13]. During latency LANA actively represses transcription of the KSHV lytic reactivator ORF50 gene (YP001129401) as well as several other lytic genes [14]–[18].

Viral cyclin (v-cyclin; U79416) is a latent KSHV gene that is transcribed from the same promoter element as LANA [19]–[21]. v-cyclin is structurally similar to cellular D-type cyclins and forms an active kinase complex with cellular CDK6 kinase (NM001259). The v-cyclin-CDK6 kinase phosphorylates not only the pRb protein (M15400), a common substrate of the cellular cyclin D-CDK6 complex, but also a large repertoire of unique cellular substrates such as p27KIP1 (AF480891), p21CIP1 (U03106), ORC-1 (U40152), CDC6 (U77949), caldesmon (M64110), and Bcl-2 (M14745) (reviewed in [22]–[24]).

We have previously demonstrated that ectopic expression of v-cyclin causes NPM redistribution from the nucleolus to the nucleoplasm [25]. Here we have addressed the functional relationship of v-cyclin and NPM in endothelial cells and naturally KSHV-infected PEL cells.

## Results

### Phosphorylation of NPM on Thr199 is dependent on v-cyclin-CDK6

To explore a functional relationship of the v-cyclin-NPM association, previously noted by us [25], in patient derived PEL cell lines we size fractionated BC-3 cell lysates and assayed for elution of NPM and v-cyclin. NPM and v-cyclin were found to co-elute at 180–110 kDa (Figure S1A; fractions marked 1–4). Subsequently, v-cyclin containing complexes were purified from these fractions and analyzed for co-precipitated CDK6 and NPM (Figure S1B), and assayed for in vitro kinase activity (Figure S1C). As shown by us before [26], v-cyclin-associated kinase activity was detected toward p27KIP1 and p21CIP1. Interestingly, v-cyclin associated kinase activity was also observed towards a 37 kDa protein most notably in fraction 2 and to a lesser extent in 3, which co-migrated with NPM (Figure S1C). These results suggested that v-cyclin associated kinase phosphorylates co-purified NPM in BC-3 PEL cells.

The notion that v-cyclin phosphorylates NPM in patient derived PEL cells was supported by recent identification of NPM Thr199 CycE-Cdk2 phosphorylation site as a target of v-cyclin-CDK6 in vitro [27]. To confirm that NPM is phosphorylated by v-cyclin on Thr199 in our experiments, U2OS cells were transfected with expression vectors for Myc-tagged v-cyclin or a vector control together with eGFP-tagged wild-type NPM (eGFP-NPM), or its phosphorylation site mutant T4A, with threonine to alanine mutations of NPM codons of 199, 214, 234, 237, and mutant T3A in which all but threonine 199 is replaced by alanine [28]. Phosphorylation of NPM was analyzed by immunoblotting using phospho-NPM antibody (pNPM Thr199), which recognizes NPM phosphorylated on its CDK2 (NM001798), phosphorylation site at threonine 199 [29]. Phosphorylation of NPM was only detected in cells co-transfected with v-cyclin and the eGFP-NPM retaining an intact Thr199 site (i.e. eGFP-NPM or eGFP-NPM T3A; Figure S1D).

**Figure 1:**
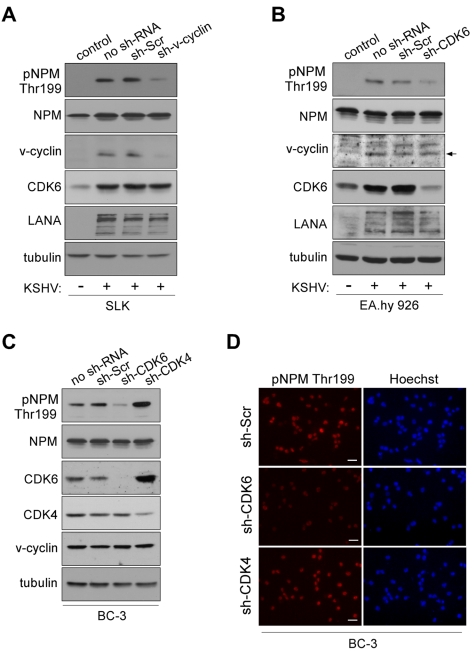
NPM phosphorylation is dependent on v-cyclin-CDK6 activity. (A) Western blotting of whole cell extracts of rKSHV.219-infected SLK cells transduced with control sh-RNA (sh-Scr) or sh-RNA against v-cyclin (sh-v-cyc) expressing retroviruses. The first lane depicts non-infected, control SLK cells. Filters were probed with antibodies against pNPM (Thr199), total NPM, v-cyclin, CDK6 and LANA. Tubulin served as a loading control. (B) Western blot analysis of rKSHV.219-infected EA.hy926 cells expressing sh-RNAs for control (sh-Scr) or CDK6 (sh-CDK6). The first two lanes show cells in the absence (control) or presence (no sh-RNA) of latent KSHV infection. Whole cell extracts were subjected to SDS-PAGE followed by immunoblotting for pNPM (Thr199), total NPM, v-cyclin, CDK6, LANA and tubulin for loading control. Arrow indicates the position of the v-cyclin band. (C) Western blot analysis of whole cell extracts of BC-3 cells expressing control sh-Scr, sh-CDK6 or sh-CDK4. The first lane shows untreated control BC-3 cells (no sh-RNA). The immunoblots were probed with antibodies against pNPM (Thr199), total NPM (NPM), CDK6, CDK4 and tubulin. (D) NPM phosphorylation in BC-3 cells expressing sh-Scr, sh-CDK4 or sh-CDK6 (left panels). Cells were stained by anti-pNPM (Thr199) and nuclei were counterstained with Hoechst. Scale bar, 20 µm.

To gain more insight into the biological relevance of v-cyclin induced phosphorylation on NPM, we next analyzed NPM phosphorylation in non-infected and KSHV-infected cells. To this end, SLK and EA.hy926 endothelial cells were infected with a recombinant KSHV, rKSHV.219 [30], and analyzed for phosphorylation of NPM by immunoblotting with the pNPM Thr199 antibody. Infection with rKSHV.219 induced prominent phosphorylation of NPM on threonine 199 in both cell lines, which interestingly, was accompanied by an increase in CDK6 protein levels (Figure 1A and B). NPM phosphorylation was detectable only at very low levels in uninfected cells (Figure 1; control lanes, see Figure S2 for longer exposure).

To demonstrate that NPM phosphorylation in KSHV-infected cells was dependent on v-cyclin, we silenced v-cyclin expression in the rKSHV.219-SLK cells using retrovirus-mediated RNA interference. We chose to use rKSHV.219-SLK cells instead of PELs since it has been reported that silencing of v-cyclin in PELs leads to cell death [31]. To this end, rKSHV.219-SLK cells were transduced with retrovirus expressing sh-RNA against v-cyclin (sh-v-cyclin) or a scrambled control sh-RNA (sh-Scr). Silencing of v-cyclin expression was confirmed by immunoblotting after 48 hours, and resulted in a 78% decrease in v-cyclin (Figure 1A). Phospho-NPM signal was markedly attenuated (4.8-fold) in cells expressing the sh-v-cyc as compared to cells expressing the sh-Scr, confirming that v-cyclin is required for NPM Thr199 phosphorylation in KSHV-infected endothelial cells (Figure 1A, top panel).

V-cyclin is transcribed from a common transcription start site for three latency associated genes (v-cyclin, v-FLIP (U90534), and LANA) [32]. The resulting transcript is spliced to yield two messages, one tricistronic encoding LANA, v-cyclin, and v-FLIP and one bicistronic message encoding v-cyclin and v-FLIP. The bicistronic transcript is more abundant of the two [20]. Therefore, targeting of v-cyclin by RNAi results in concurrent depletion of v-FLIP [31]. To rule out the possibility that the inhibition of NPM phosphorylation in sh-v-cyclin expressing cells is due to the loss of v-FLIP, rKSHV.219-SLK cells expressing sh-v-cyclin or sh-Scramble were reconstituted for v-FLIP by transducing them with a retrovirus expressing v-FLIP (v-FLIP-pBMN) or empty vector (pBMN). Consistent with the bicistronic nature of the v-cyclin transcript, expression of sh-v-cyclin reduced the level of v-cyclin and v-FLIP transcripts to 27% and 48%, respectively, of the levels in control cells (sh-v-cyclin -; Figure S3A). This change was accompanied by significant reduction in NPM Thr199 phosphorylation in the sh-v-cyclin cells expressing either pBMN (v-FLIP -) or v-FLIP-PBMN (v-FLIP +), suggesting that reconstitution of v-FLIP had no effect on NPM phosphorylation (Figure S3B). This data further underscores the importance of v-cyclin in phosphorylation of NPM in KSHV-infected endothelial cells. As inhibition of v-cyclin or v-FLIP has been shown to induce apoptosis in PEL cells [31],[33],[34] we analyzed whether RNAi for v-cyclin resulted in changes in the cell cycle or increased cell death. To this end expression of cyclin A (U66838) as a relevant S-phase marker and apoptosis by TUNEL assay were determined. No significant differences in the cyclin A levels (Figure S3B) or in TUNEL positivity (data not shown) were observed in the sh-v-cyclin cells as compared to the sh-Scr cells.

Previous studies have shown that CDK6 is the major kinase partner of v-cyclin in PEL cells [35],[36]. We therefore sought to confirm that also CDK6 is required for NPM phosphorylation in KSHV-infected cells. To this end, we depleted CDK6 in the rKSHV.219-infected endothelial EA.hy926 cells, and, in addition, CDK4 (M14505) and CDK6 (NM 001259) in BC-3 cells using lentivirus-mediated RNA interference. Cells stably expressing sh-RNAs specific for the aforementioned kinases or control sh-RNA (sh-Scr) were first assayed for the levels of respective kinases. Immunoblotting analysis indicated 80% decrease of CDK6 in EA.hy926, and a 98% and a 60% decrease of CDK6 and CDK4 in BC-3, respectively (Figure 1B and 1C). The specificity of silencing was also controlled by reciprocal immunoblotting of the kinases in BC-3 cells (i.e. anti-CDK6 for sh-CDK4-expressing cells and vice versa; Figure 1B). Silencing of CDK4 resulted in a 3.6-fold compensatory increase in CDK6. This is in accordance with previous studies that have illustrated that CDK4 activity is dispensable for most cell types, mostly due to the compensation by the highly related CDK6 [37]–[39]. Suppression of v-cyclin associated kinase activity consequent to CDK6 silencing was confirmed by an *in vitro* kinase assay in BC-3 cells (Figure S4). Depletion of CDK4/6 neither induced a cell cycle arrest (Figure S5) nor increased apoptosis (data not shown) in the sh-CDK expressing BC-3 cells. Importantly, we observed that NPM phosphorylation was significantly reduced in rKSHV.219-EA.hy926 (4.8-fold) and BC-3 cells (3.7-fold) silenced for CDK6 as compared to cells stably expressing the scrambled control (Figures 1B–D) or sh-CDK4 (Figures 1C and 1D). In contrast, concomitant with an increase in CDK6 level in the CDK4-silenced cells, the phosphorylation of NPM was increased by 1.8-fold. Taken together, the results indicate that v-cyclin phosphorylates NPM on Thr199 by activating CDK6 in the KSHV-infected endothelial and BC-3 cells, which are both biologically relevant cell types in KSHV-infections and malignancies.

### NPM interacts with LANA in a v-cyclin dependent manner

**Figure 2:**
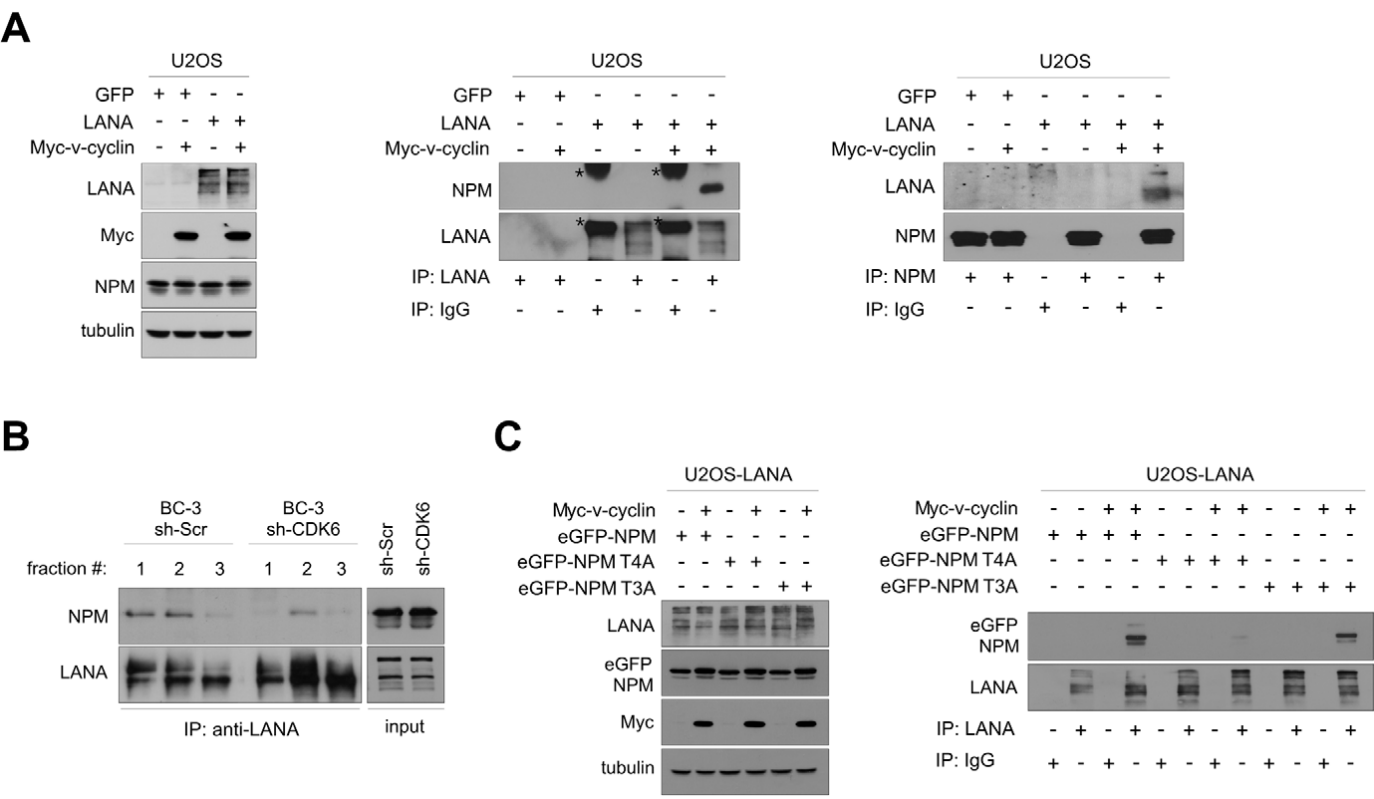
NPM interaction with LANA is dependent on Thr199 phosphorylation of NPM by v-cyclin-CDK6. (A) U2OS cells stably expressing GFP or LANA were transfected with an expression vector for Myc-v-cyclin or with an empty vector as indicated. Cell extracts (10% of immunoprecipitates) were resolved by SDS-PAGE (left panel) or were immunoprecipitated with anti-LANA (middle panel) or anti-NPM antibodies (right panel). IgG immunoprecipitations were included as controls. Immunoblotting was performed with anti-NPM and –LANA as indicated. Asterisks indicate the nonspecific crossreactivity detected with NPM and LANA antibodies. (B) Gel filtration fractions marked 1-3 (ca. 700-440 kDa) of BC-3 cells expressing sh-Scr or sh-CDK6 (as described in Figure 1C) were immunoprecipitated with anti-LANA antibodies, separated by SDS-PAGE and followed by immunoblotting with antibodies against NPM and LANA. Input (10%) is indicated on the right. (C) U2OS cells stably expressing LANA were transfected with the Myc-v-cyclin or empty vector, and the NPM expression constructs for wt (eGFP-NPM) and phosphorylation site mutants eGFP-NPM T4A and T3A as shown on top. Cell extracts (10% of immunoprecipitates) were analyzed by SDS-PAGE (left panel) for the input or were immunoprecipitated with antibodies against LANA or control rat IgG (right panel). The immunoblots were probed with with anti-GFP and –LANA as indicated.

A study by Si et al. [40] reported that NPM associates with LANA and the terminal repeats of the KSHV genome in PEL cells. That LANA and NPM interact is supported by gel filtration chromatography and co-immunoprecipitation analysis of LANA and NPM in BC-3 cells (Figure S6 and [37]). Given that NPM is detected in complexes with two KSHV latent proteins, which are transcribed from a common promoter upstream of the LANA gene [19],[32], we considered it reasonable to test whether v-cyclin affects LANA-NPM association. We transfected U2OS cells stably expressing LANA or GFP (Sarek et al., unpublished data) with a Myc-tagged v-cyclin expression vector (Myc-v-cyclin) or with an empty vector as a control. After 48 hours, whole cell extracts were collected and subjected to immunoprecipitation with anti-LANA or -NPM antibodies followed by reciprocal immunoblotting. This revealed that co-expression of v-cyclin facilitated association of NPM with LANA (Figure 2A; middle and rightmost panels). To further address the NPM-LANA interaction in naturally KSHV-infected PEL cells, and the dependency of this interaction on CDK6, the sh-Scr and sh-CDK6 BC-3 cells shown in Figure 1B were subjected to gel filtration chromatography to assess for LANA and NPM co-elution. NPM and LANA were found to co-elute and co-precipitate in fractions marked 1–3, corresponding to ca. 700-440 kDa (Figure S7). Interestingly, silencing of CDK6 reduced co-precipitation of NPM and LANA in these fractions (Figure 2B), further indicating that the v-cyclin-CDK6 kinase activity supports the NPM-LANA interaction.

We then examined the requirement of NPM phosphorylation on Thr199 for NPM-LANA interaction, by performing anti-LANA immunoprecipitation from cell extracts of U2OS cells expressing LANA and transfected with expression vectors for Myc-v-cyclin, empty vector control, and the eGFP-tagged NPM wt or its phosphosite mutant constructs used in Figure S1D. As shown in Figure 2C (right panel), LANA associated with the eGFP-tagged NPM wt and T3A mutant in the presence of v-cyclin, while interaction with the T4A mutant was severely compromised. Together these results indicate that the phosphorylation of Thr199 on NPM by v-cyclin–CDK6 is needed for LANA-NPM interaction.

### Interaction of NPM and LANA is affected by acetylation

**Figure 3:**
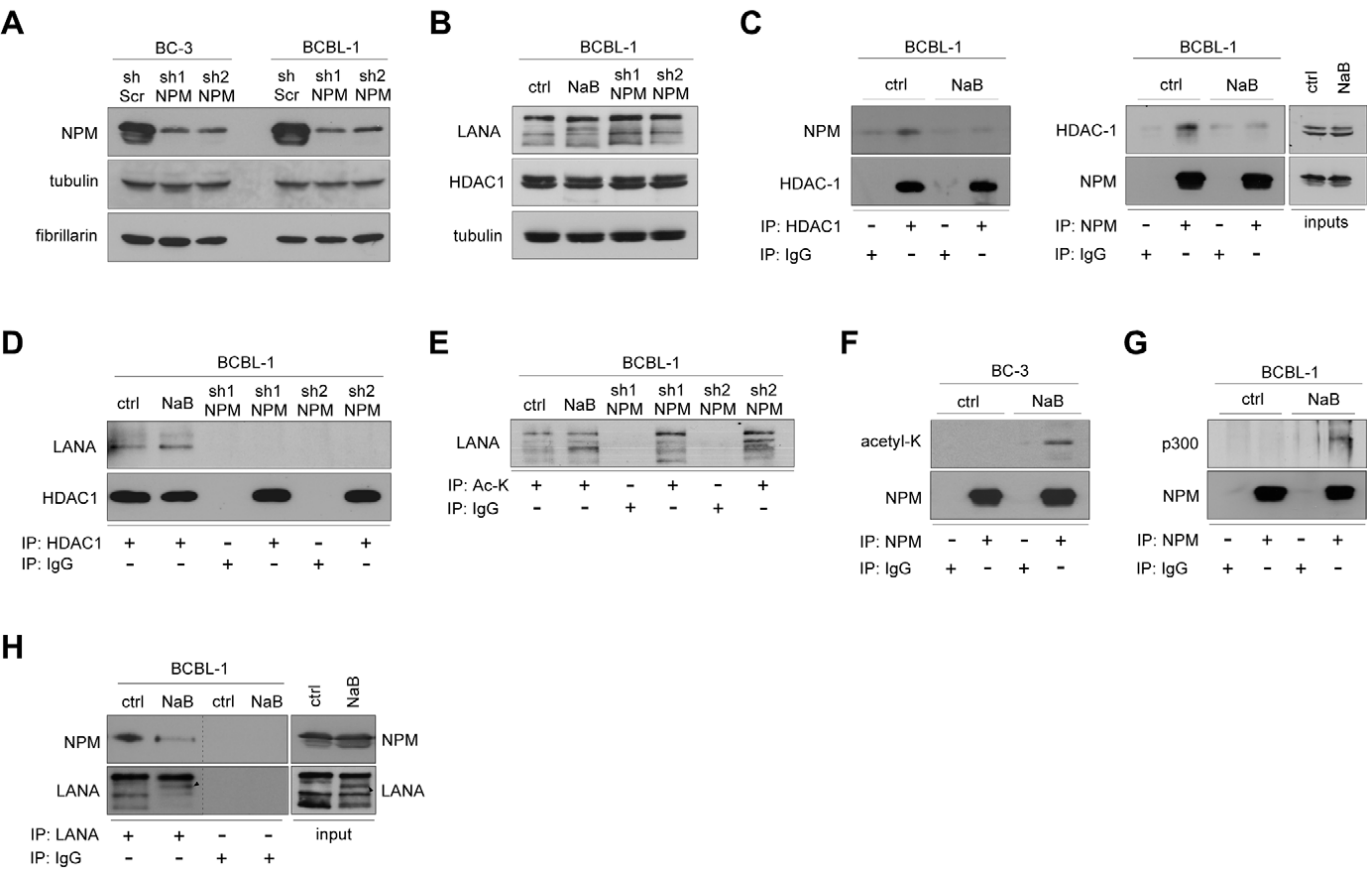
Acetylation regulates NPM interaction with LANA. (A) Whole cell extracts of BC-3 and BCBL-1 cells stably expressing sh-Scr, or two different constructs of sh-RNA specific for NPM (sh1-NPM and sh2-NPM) were analyzed by immunoblotting with anti-NPM antibodies. Fibrillarin and tubulin were used as loading controls. (B) Extracts of BCBL-1 cells untreated (ctrl), NaB treated (NaB), or cells stably expressing sh1-NPM or sh2-NPM were separated by SDS-PAGE followed by immunoblotting with antibodies against LANA, HDAC1 and tubulin. These samples represent the input (10%) for panels D and E. (C) Whole-cell extracts of ctrl and NaB-treated BCBL-1 cells from B were reciprocally immunoprecipitated using anti-HDAC1 or anti-NPM antibodies. Rabbit or mouse normal IgG (IgG) served as a control for immunoprecipitation. Protein complexes were resolved by SDS-PAGE and followed by immunoblotting with anti-NPM and anti-HDAC1 antibodies. Input (10%) is shown on the right. (D) Whole cell extracts of BCBL-1 cells in *B* were immunoprecipitated with anti-HDAC1 antibodies. Normal rabbit IgG was used as a negative control. The immunocomplexes were separated by SDS-PAGE followed by immunoblotting with antibodies against LANA and HDAC1. (E) Whole cell extracts of BCBL-1 cells used in panel B were immunoprecipitated with anti-acetyl lysine antibody and analyzed for indicated proteins by immunoblotting. Rabbit IgG served as a negative control for immunoprecipitation. (F) BC-3 cells were treated with vehicle (ctrl) or 1 mM sodium butyrate (NaB) for 24 hours. Whole-cell extracts were subjected to immunoprecipitation using anti-NPM (NPM) or normal mouse control IgG (IgG). The samples were separated by SDS-PAGE and followed by immunoblotting with anti-acetyl-K lysine and anti-NPM antibodies. (G) Whole-cell extracts of ctrl and NaB-treated (24 hours) BCBL-1 cells from B were subjected to immunoprecipitation using anti-NPM (NPM) or normal mouse control IgG (IgG). The samples were separated by SDS-PAGE and followed by immunoblotting with anti-p300 and anti-NPM antibodies. (H) Whole cell extracts of BCBL-1 cells untreated (ctrl), or NaB-treated (NaB), were immunoprecipitated with anti-LANA antibodies (left panel). Protein complexes were resolved by SDS-PAGE and followed by immunoblotting with anti-NPM and -LANA antibodies. Input (10%) is indicated in the right panel. Arrowheads indicate the position of possibly acetylated LANA. For the purpose of clarity of the data Western blots lanes (derived from the same SDS-PAGE gel) were reorganized as indicated by black dashed bars.

To explore the role of NPM in KSHV pathobiology we first silenced NPM in BC-3 and BCBL-1 PEL cell lines using lentivirus-mediated RNA interference. NPM protein level was decreased by 77% and 80% in BC-3 and 83% and 80% in BCBL-1 cells stably expressing two different sh-RNAs for NPM, respectively (sh1-NPM and sh2-NPM) as compared to cells expressing a control sh-Scr (Figure 3A). Fibrillarin (AC005393), another nucleolar protein, and tubulin were used as loading controls and were unaffected. There were no major effects on mitotic activity and proliferation (data not shown), or on nucleolar morphology based on localization of fibrillarin (Figure S8), or phase contrast in the selected population of cells silenced for NPM (data not shown). Furthermore, no obvious change was observed for LANA levels in cells expressing sh-RNAs against NPM (as shown for BCBL-1 cells in Figure 3B).

KSHV is predominantly latent in PEL cells, but can be induced to lytic replication phase with chemical HDAC inhibition. NPM was recently reported to recruit HDAC and to exert its repressive effect on transcription by inducing a change in local chromatin structure [41]. To address the function of NPM in PEL cells in this context, we first analyzed the association of NPM and LANA with HDAC1 (D50405) by immunoprecipitation experiments. In agreement with the recently reported role of NPM as an HDAC recruiter, NPM was consistently present in reciprocal co-immunoprecipitations with HDAC1 in latent (uninduced) BCBL-1 cells (Figure 3C) and BC-3 cells (data not shown). Consequently, we tested whether this interaction is affected by inhibition of HDAC activity with sodium butyrate (NaB) that leads to induction of lytic phase (reactivation). The association between NPM and HDAC1 was greatly diminished upon NaB treatment while their protein levels remained unchanged (Figure 3C). Interestingly, we found that LANA complexed with HDAC1 in the latently infected cells and even after HDAC inhibition by NaB (Figure 3D). This interaction, however, was completely abolished upon NPM silencing, suggesting that NPM facilitates interaction of LANA with chromatin modifiers (Figure 3D).

NaB treatment has been shown to increase LANA acetylation and to diminish LANA interactions with chromatin, Sp1 transcription factor, and the early lytic ORF50/RTA promoter [42]. Given that NPM silencing reduced the association of LANA with HDAC1, we decided to assess for acetylation of LANA in both NPM silenced and NaB-treated BCBL-1 cells. To this end we immunoprecipitated cellular extracts with antibody against acetylated lysine (Ac-K) or control IgG antiserum, followed by immunoblotting with anti-LANA antibody. As shown in Figure 3E treatment with NaB as well as silencing of NPM expression resulted in appearance of new bands in the subsequent LANA immunoblot suggesting of an increase in the acetylation of LANA [42]. However, we cannot exclude the possibility that we were detecting other acetylated proteins associated with LANA. Acetylation of NPM by the histone acetyltransferase p300 (NP001420) has been reported to disrupt the nucleosomal structure [43]. We therefore analyzed the level of acetylation on NPM in the NaB-treated BC-3 cells, and found that it was increased upon HDAC inhibition (Figure 3F). In line with this, p300 was co-immunoprecipitated with NPM from extracts of BCBL-1 cells treated with NaB (Figure 3G).

Protein acetylation is one possible mechanism modulating the interaction between LANA and NPM. Thus, we wanted to test whether this interaction is altered by NaB. Whole cell extracts of BCBL-1 cells treated with NaB (1 mM) or vehicle for 24 hours were subjected to immunoprecipitation with anti-LANA followed by immunoblotting for NPM. Intriguingly, we found that NPM association with LANA was abolished in cell extracts pretreated with NaB (Figure 3H). Given that NaB treatment leads to induction of viral reactivation, we considered it reasonable to rule out the possibility that expression of lytic genes would affect the LANA-NPM association. We have recently demonstrated that Pim-1 kinase (NM002648) is a critical regulator of KSHV reactivation, and that depletion of Pim-1 expression by RNAi leads to inhibition of early steps of viral reactivation [44]. To this end we transfected BCBL-1 cells with siRNA oligonucleotides specific for Pim-1 (Pim-1 siRNA) or with a non-targeting control siRNA (Scr siRNA). Cells were subjected to 1 mM NaB or vehicle treatment 48 hours post-transfection and lysates were collected after another 24 hours to confirm silencing of Pim-1 (Figure S9A, left panel). The inhibition of viral reactivation by Pim-1 depletion was confirmed by quantitative real-time PCR (Figure S9B) and immunobloting with vIL-6 (AAB61701) (Figure S9C). Importantly, NPM association with LANA was still abolished by inhibition of HDAC activity in the Pim-1 depleted cell extracts (Figure S9A, right panel), supporting that the NPM-LANA interaction was disrupted due to inhibition of de-acetylation, and not due to induction of lytic gene expression.

### Depletion of NPM leads to induction of a complete lytic replication cascade in PEL cells

**Figure 4:**
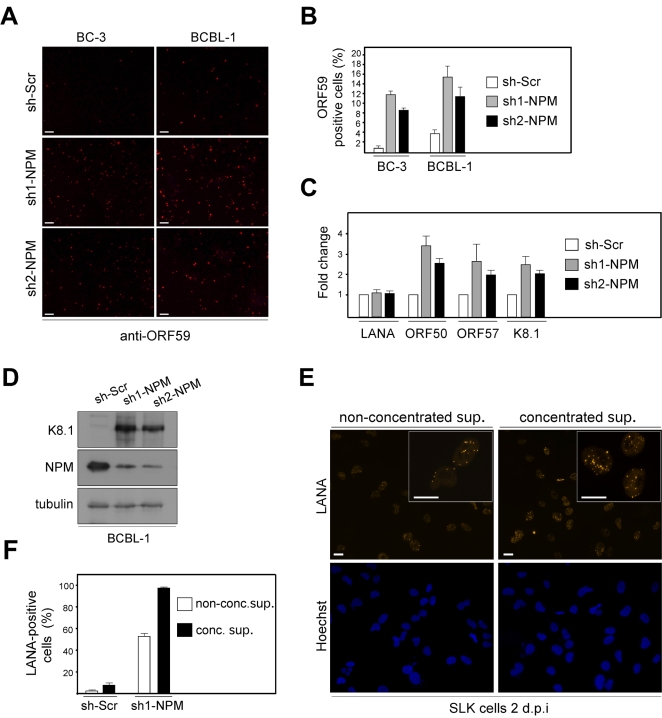
NPM depletion triggers induction of the full lytic replication cascade in PEL cells. (A) BC-3 and BCBL-1 cells transduced with the non-target sh-Scr, sh1-NPM, or sh2-NPM were stained with antibodies against an early lytic marker ORF59 (anti-ORF59) and analyzed by indirect immunofluorescence. Images are representative of two independent experiments. Scale bar = 100 µm. (B) Quantification of the ORF59 positive BC-3 and BCBL-1 cells in panel A. Values are means of two independent experiments ± SD. (C) Total RNA from the BCBL-1 cells used in panel A were assayed for the abundance of LANA, ORF50, ORF57, and K8.1 transcripts by real-time quantitative PCR. The relative abundances of the latent and lytic transcripts in NPM depleted cells were compared to control sh-Scr infected cells. Transcripts for human beta-actin were used to normalize the results. Data is representative of two independent experiments ± SD. (D) Whole cell extracts of the BCBL-1 cells used in A were analyzed by immunoblotting with anti-K8.1, anti-NPM and anti-tubulin antibodies. (E) Supernatant from BCBL-1 cells transduced with sh1-NPM were collected and concentrated (conc. sup.), or used unconcentrated (non-conc. sup.) to infect naive SLK cells. Two days postinfection, establishment of latency was monitored by LANA expression in SLK cells by immunofluorescence microscopy. Images are representative of two independent experiments. Scale bar = 20 µm. Insets highlight representative cells at higher magnification. (F) Quantification of the number of LANA positive SLK cells two days post infection either with non-concentrted supernatant or concentrated supernatant derived from BCBL-1 cells transduced with control sh-Scr or sh1-NPM. Data is representative of two independent experiments ± SD.

As silencing of NPM seemed to induce an increase in LANA acetylation in PEL cells, an event previously linked to viral reactivation, we next addressed the expression of lytic replication markers in the NPM silenced BC-3 and BCBL-1 cells. Cells stably expressing a non-target control sh-RNA (sh-Scr), sh1-NPM, and sh2-NPM were analyzed by immunofluorescence using antibodies against an early lytic marker ORF59 (YP001129416). In both cell lines, depletion of NPM expression led to a significant increase in ORF59 positive cells as compared to the sh-Scr expressing control cells (shown for BCBL-1 in Figures 4A and 4B). Viral reactivation in sh1-NPM and sh2-NPM expressing BCBL-1 cells was further confirmed by quantitative real-time PCR (qRT-PCR) for the early lytic genes ORF50 and ORF57 (YP001129410) as well as for a late lytic gene K8.1 (Figure 4C).

Reactivation of the complete lytic cascade of KSHV results in production of infectious progeny virions. We therefore wanted to investigate whether NPM silencing would lead to production of infectious virions in PEL cells. To this end, BCBL-1 cells stably expressing sh1-NPM, sh2-NPM, or sh-Scr were analyzed by immunoblotting for expression of the late lytic capsid glycoprotein K8.1 (AF068829), nine days post-transduction. Expression of K8.1 was induced in cells depleted for expression of NPM with sh1- or sh2-NPM, but not in cells expressing the control sh-Scr (Figure 4D). To measure production of infectious KSHV particles upon NPM silencing, supernatants from sh1-NPM or sh-Scr expressing BCBL-1 cells were collected six, nine and 12 days post lentiviral transduction, pooled, and used directly or after concentration by ultracentrifugation to infect naive SLK cells. Infection of target cells was monitored by immunofluorescence with anti-LANA antibodies. At 48 hours after infection, 50–90% of the target SLK cells displayed the typical speckled signal from LANA with numerous dots per nucleus thus confirming that silencing of NPM expression led to induction of the full lytic replication cascade and production of infectious virions (Figure 4E and F).

### Phosphorylation of NPM correlates with KSHV latency

**Figure 5:**
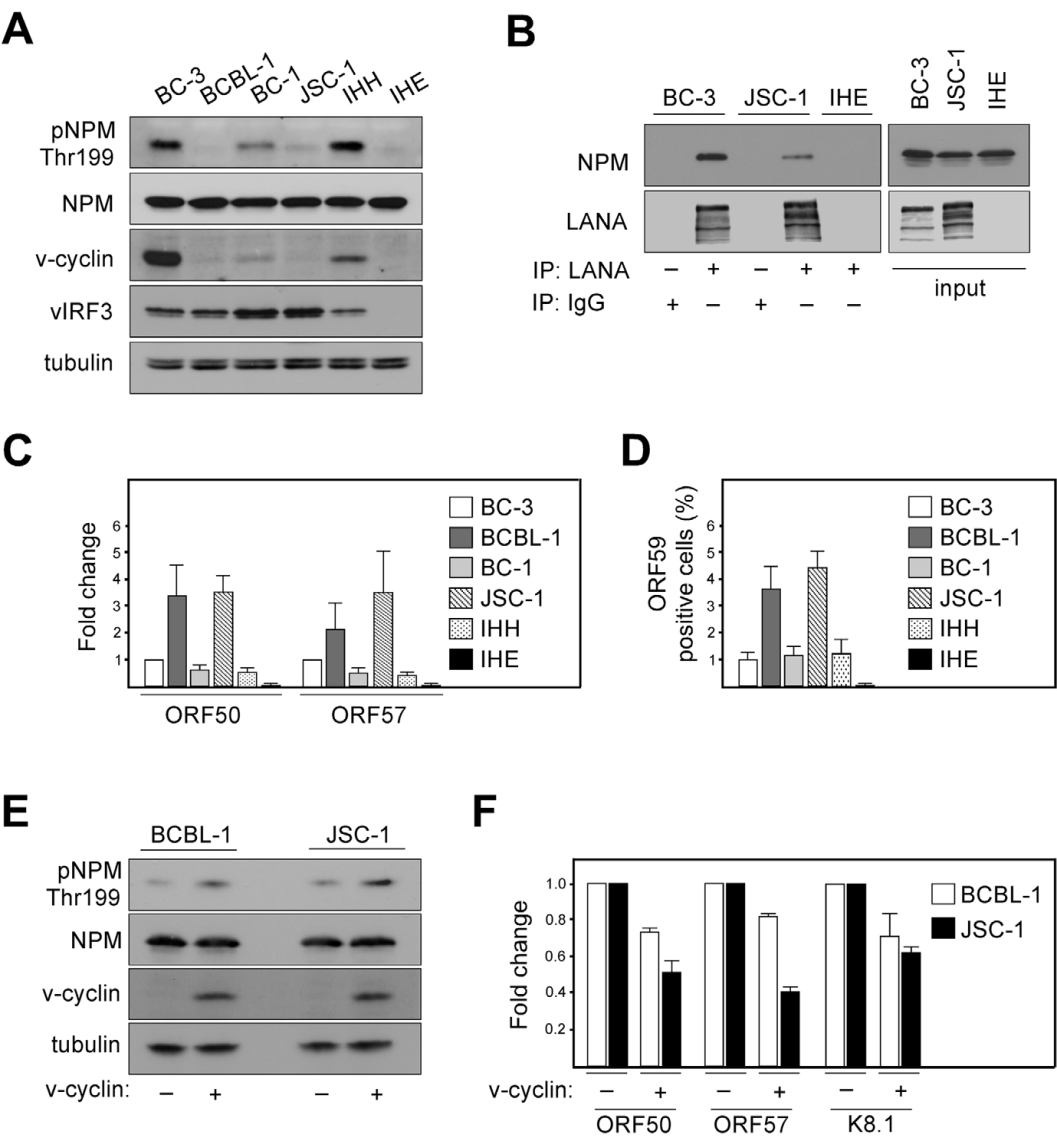
Phosphorylation of NPM correlates with KSHV latency. (A) Whole-cell extracts of KSHV-positive (BC-3, BCBL-1, BC-1, JSC-1, IHH) and KSHV-negative (IHE) cell lines were subjected to SDS-PAGE followed by Western blotting and analyzed for pNPM Thr199, total NPM (NPM), v-cyclin and vIRF3. Tubulin was used as a loading control. (B) Cell extracts of BC-3, JSC-1 and IHE cells were immunoprecipitated using anti-LANA or rat IgG as a control. Immunocomplexes were resolved by SDS-PAGE and analyzed by Western blotting with anti-LANA and anti-NPM antibodies. Input (5%) is indicated on the right. (C) Total RNA from cells in panel A was assayed for the abundance of ORF50 and ORF57 transcripts by real-time quantitative PCR. Transcripts of human β-actin were used to normalize the values. Data is representative of three independent experiments ± SD. The relative abundances of the lytic transcripts were compared to those in BC-3 cells, which was set to one. (D) Quantification of the ORF59 positive cells used in panels A and C. Values are means of three independent experiments ± SD. (E) BCBL-1 or JSC-1 cells were acutely transduced with retroviruses expressing GFP (v-cyclin -) or v-cyclin (v-cyclin +). Whole-cell extracts were resolved by SDS-PAGE at 48 hours post-transduction and immunoblotted for pNPM Thr199, total NPM (NPM), and v-cyclin. Tubulin served as a loading control. (F) Total RNAs from cells in panel E were assayed for the abundance of ORF50, ORF57 and K8.1 transcripts by real-time quantitative PCR. Transcripts of human β-actin served as an endogenous control. The relative abundances of the lytic transcripts in v-cyclin over-expressing BCBL-1 and JSC-1 cells (v-cyclin +) were compared to those in the corresponding control cells (v-cyclin -), which were set as one. Data is representative of two independent experiments ± SD.

Considering the essential role of LANA in KSHV latency and suppression of lytic viral transcription, as well as the observation that v-cyclin promotes LANA-NPM interaction, we sought to address whether there is a correlation between NPM Thr199 phosphorylation, v-cyclin expression, and the extent of spontaneous lytic replication in four different patient-derived KSHV-infected PEL lines (BC-3, BCBL-1, BC-1, JSC-1), IHH (a KSHV-positive lymphoblastoid cell line), and IHE (a KSHV-negative cell line). To this end, cell extracts were analyzed by immunoblotting for expression of pNPM Thr199, total NPM (NPM), v-cyclin, and another latent KSHV protein vIRF3 as a control. Interestingly, we found an apparent correlation with the extent of NPM phosphorylation and v-cyclin expression in the KSHV-infected lymphoid cells (Figure 5A). In accordance with our finding that phosphorylation of NPM is needed for the interaction between LANA and NPM (Figure 2), the LANA-NPM interaction was diminished in JSC-1 PEL cells, which had a very low level of NPM phosphorylation, as compared to BC-3 cells with highly phosphorylated NPM and prominent LANA-NPM co-precipitation (Figure 5B). To examine whether NPM phosphorylation correlates with the level of spontaneous, un-induced viral reactivation, we performed qRT-PCR for expression of the early lytic transcripts ORF50 and ORF57 in the KSHV-infected PEL cells (Figure 5C). Reactivation was also analyzed by immunofluorescence using antibodies against the early lytic marker ORF59 (Figure 5D). Intriguingly, we found that the cells with lower phospho-NPM levels had higher level of spontaneous expression of the lytic markers compared to the cells with elevated phospho-NPM, suggesting that NPM is a critical regulator of latency in KSHV-infected lymphoid cells.

To obtain further evidence about the correlation between, v-cyclin expression levels, extent of NPM phosphorylation on Thr199, and spontaneous viral reactivation we over-expressed v-cyclin in BCBL-1 and JSC-1 cells using retroviruses expressing v-cyclin and GFP (KpBMN) or GFP (pBMN) as a control. Exogenous expression of v-cyclin (determined by the number of GFP positive cells) was achieved in 17% and 20% of BCBL-1 and JSC-1 cells, respectively (data not shown), and confirmed by Western blotting at 48 hours post-transduction (Figure 5E). As shown in Figure 5E, NPM phosphorylation on Thr199 increased about 1.8-fold in cells over-expressing v-cyclin as compared to cells infected with the control (v-cyclin + and -, respectively) as evaluated from the luminescence signal, and analyzed by Image J software. In accordance with the increase of pNPM Thr199 levels, qRT-PCR analysis demonstrated a 1.5 to 1.7-fold reduction (in average) in the spontaneous expression of the lytic transcripts ORF50, ORF57 and K8.1 in cells over-expressing v-cyclin (Figure 5F; v-cyclin +). Taken together, these results further support the role of v-cyclin mediated phoshorylation of NPM in controlling spontaneous viral reactivation in PEL cells.

### NPM is phosphorylated in KS tumors

**Figure 6:**
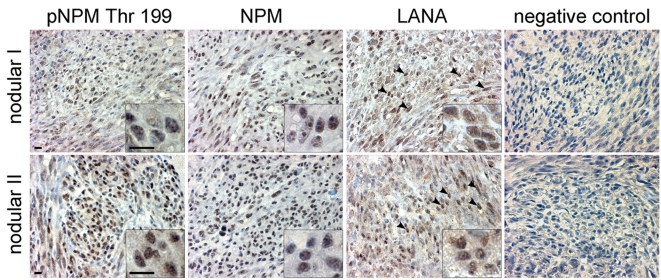
KS tumors express NPM phosphorylated on Thr199. Paraffin-embedded sections of two representative late stage (nodular) KS skin lesions (nodular I and II) were stained for pNPM Thr199, total NPM (NPM) and LANA. Images were captured at 63X magnification. LANA signal appears as brown nuclear staining (arrowheads). Insets highlight representative cells at higher magnification. Omission of the primary antibody served as a negative control (negative control). Scale bars; 10 µm.

Our results suggest that KSHV infection and more specifically the activity of v-cyclin-CDK6 is responsible for the phosphorylation of NPM on Thr199 in latently infected cells. To determine whether NPM is phosphorylated also in KS tumors we stained primary cutaneous lesions of KS (n = 6) with antibodies against pNPM Thr199 and total NPM. Immunohistochemistry for LANA confirmed KSHV infection and defined the tumor area (Figure 6, LANA). Sections of paraffin-embedded BC-3 cells stably expressing sh-CDK6 or control (sh-Scr) were used to verify the specificity of anti-pNPM Thr199 staining (data not shown). NPM phosphorylation was observed in all of the KS lesions analyzed (Figure 6), suggesting a role for NPM phosphorylation in KSHV pathogenesis.

## Discussion

Latency is the predominant mode of viral persistence in KS and PEL tumors, and is considered to have a fundamental impact on KSHV tumorigenesis. In this study, we show that NPM is phosphorylated in KSHV-infected cells by v-cyclin-CDK6. In addition, we show that NPM is phosphorylated in primary human KS tumors. Our study demonstrates the functional interaction between v-cyclin and NPM in a biologically relevant system, and establishes NPM as a substrate for the v-cyclin activated CDK6 in KSHV-infected endothelial cells and PEL cells. Our findings indicate that phosphorylation of NPM is essential for its association with LANA, and thus establishes the first functional link between the latent proteins v-cyclin and LANA. Furthermore, we demonstrate that NPM is a critical key regulator in KSHV latency.

Our data indicates that in latently KSHV-infected cells NPM facilitates interaction of LANA with HDAC1 (Figure 3D) and the core histones (data not shown). This is in accordance with the recent finding identifying NPM as a HDAC recruiter involved in transcriptional repression during differentiation [41], and with earlier data showing that HDACs interact directly or indirectly with viral sequences to inhibit lytic gene expression [45]. A study by Lu et al. (2006) has shown that LANA-mediated repression of viral lytic transcription is mediated by LANA binding to the lytic reactivator ORF50/RTA promoter, which is de-repressed by acetylation of LANA [42], probably due to acetylation disturbing the association of LANA with the chromatin modifiers at the ORF50 promoter. Here our results demonstrate that in cells depleted for NPM LANA is not anymore associated with HDAC1 (Figure 3D). Moreover, depletion of NPM led to an increase in the acetylation of LANA (Figure 3E), and induction of the complete lytic replication cascade (Figure 4). In line with these results, inhibition of HDAC activity by NaB is a widely used mechanism to induce KSHV reactivation. Further supporting the role of acetylation in the LANA-NPM interaction, NaB treatment led to an increase also in lysine acetylation of NPM (Figure 3F), and to dissociation of the NPM-LANA complex (Figure 3H). Interestingly, NaB treatment did not disrupt the interaction of LANA with HDAC1 (Figure 3D), probably because the increase in LANA acetylation was achieved by chemical inhibition of the HDAC enzymatic activity rather than by interfering with its recruitment by NPM. Furthermore, it is possible that acetylation primarily modulates NPM-HDAC1 and NPM-LANA interactions in the ternary complex of NPM-HDAC1-LANA, but does not interfere with the HDAC1-LANA interaction after the recruitment of HDAC1 to the complex has occurred by phosphorylated NPM. However, if de-acetylation by HDAC-1 is chemically suppressed (by NaB) this results in an increase of NPM acetylation (Figure 3F), dissociation of HDAC1 from NPM (Figure 3C), and may allow a more stable interaction between p300 and NPM as suggested by the data in Figure 3G. Although also acetylation on LANA is elevated, this only affects its association to NPM, but not to HDAC-1 that would remain associated with LANA (Figure 3D).

NPM has also been implicated in the replication cycle of other viruses such as adeno-[46],[47], and adeno-associated viruses [48], but the detailed mechanism for the function of NPM in these viral systems is not known. By analyzing several different PEL cell lines and one in vitro KSHV-infected lymphoblastoid cell line we detected significant differences in the level of phosphorylation of NPM, but not in total NPM, which correlated with v-cyclin expression (Figure 5A). Furthermore, we found that the extent of NPM phosphorylation correlated with the low level of spontaneous reactivation in these cells.

Taken together, our study identifies a key role for NPM and its phosphorylation in regulating the KSHV latency in PEL lymphocytes and de novo infected endothelial cells. The data demonstrating a significant reduction in the phosphorylation of NPM upon silencing of CDK6 expression in KSHV-infected endothelial and lymphoid cells (Figure 1) may open up novel opportunities for developing targeted therapies for intervention and treatment of KSHV associated malignancies. Putative approaches could include development of CDK6-specific kinase inhibitors or addressing the effect of small molecule NPM inhibitors or RNA aptamers [49],[50] on disruption of KSHV latency.

## Materials and Methods

### Cell lines

BC-1, and JSC-1 PEL cell lines [51],[52] were obtained from ATCC (Manassas, VA). BC-3 and BCBL-1 cell lines [8],[53] were kindly provided by E. Cesarman (Cornell Medical College, NY), and IHH and IHE [54] by J. Haas (University of Edinburgh). SLK cells were a kind gift of T. F. Schulz (Hannover Medical School, Germany). PEL cell lines were cultured as described previously [26]. To induce KSHV lytic replication, cells were treated with 1 mM sodium butyrate (NaB; Sigma, St Louis, MO) for 24 hours. U2OS human osteosarcoma cells (ATCC) were grown as detailed in [26]. rKSHV.219-infected endothelial cells were established and maintained as described in [54]. Infected cells were grown in the presence of 1 µg/ml of puromycin for about two weeks to obtain a 100% infected cell population.

### Lenti- and retroviral production and transduction

Production of retro- and lentiviral supernatants and CDK silencing in EA.hy926 and SLK cells were performed as described earlier [55]. To silence CDKs and NPM, the PEL cells were seeded at density 5×10^5^/ml and transduced in a 50 ml culture flask using 2 ml lentiviral supernatants in the presence of 8 µg/ml polybrene (Sigma). 24 hours after transduction, the culture was replenished with fresh media, and cells were kept for 48 hours, after which they were subjected to selection with 3.5 µg/ml puromycin (sh-NPM, sh-Scr) or 300 µg/ml hygromycin (sh-CDK4, sh-CDK6, sh-Scr).

For an acute depletion of v-cyclin expression in the rKSHV.219-SLK cells, the cells were spin-transduced (2500 rpm; Heraeus Multifuge 3 S-R; Thermo Scientific) for 30 min at room temperature with fresh amphotropic retroviruses expressing control sh-RNA (sh-Scr) or sh-RNA against v-cyclin (sh-v-cyclin) in the presence of 8 µg/ml polybrene. Cells were then returned to 37°C, 5% CO_2_, and after 24 h of incubation viral supernatant was removed and replaced with fresh complete media. Cells were harvested for analysis 48 hours post-transduction.

U2OS osteosarcoma cells were spin-transduced as described above with GFP- or LANA-expressing lentiviruses in the presence of 8 µg/ml polybrene, and the cells were incubated for 48 hours. Thereafter LANA and GFP-expressing cells were cultured in the presence of 4.5 µg/ml of blasticidin (Sigma) for at least two weeks. Stable expression of the transduced proteins was assessed by immunofluorescence and Western blotting using anti-GFP or anti-LANA antibodies.

### Analysis of production of infectious virions

To study production of infectious virions upon the silencing of NPM expression, BCBL-1 cells (0.5×10^6^/ml) were transduced with sh-Scr and sh1-NPM lentiviral supernatants as described above. The medium was collected at day six, nine, and 12 post-transduction. The supernatants were cleared by centrifugation at 3,000×g for 15 min to remove cell debris and pooled together. The supernatants were concentrated at 21,000 rpm (60,000×g) for 2 hours in a Beckman SW28.1 rotor, resuspended overnight in 1 ml (1/100 of the original volume) of TNE buffer (10 mM Tris-HCl, 0.15 M NaCl, 1 mM EDTA [pH 7.8]), and used to infect naive SLK target cells.

### Protein analysis and kinase assay

Immunoblotting, immunoprecipitations, kinase assay and size exclusion chromatography were carried out as detailed [26]. For detection of the p-NPM signal by western blotting see Text S1.

### Real-time quantitative PCR

Total RNA was prepared by using the RNAeasy kit according to instructions from the manufacturer (Qiagen, Valencia, CA). RT-PCR was performed with TaqMan Reverse Transcription Reagents kit (Roche Diagnostics, Indianapolis, IN) according to manufacturer's protocol. Real-time PCR conditions and set of primers for LANA, v-cyclin, v-FLIP, ORF50, ORF57, K8.1 and human beta-actin were essentially as described previously [56],[57].

### Immunohistochemistry

Sections of paraffin-embedded KS tumors were treated for antigen retrieval by autoclaving in 10 mM sodium citrate (pH 6.0) for 2 min, and then pretreated for peroxidase blocking by incubation in 2.5% H_2_O_2_ for 30 min. Sections were processed by using Vectastain Elite ABC rabbit IgG or mouse IgG kits (Vector Laboratories, Burlingame, CA). Sections were blocked for 30 min at room temperature in normal goat serum (1∶50 in PBS) +0.3% BSA. The slides were incubated at +4°C overnight with primary antibodies to pNPM (Thr199) or total NPM diluted 1∶200 or 1∶100, respectively, in PBS +0.3% BSA followed by biotinylated anti-rabbit or anti mouse secondary antibody 1∶200 (Vectastain), respectively in 0.3% BSA in PBS for 30 min at room temperature. Signal was amplified by ABC (Vectastain) and diaminobenzidine (DAB; DakoCytomation) reaction. The slides were then counterstained with hematoxyline and mounted by using GVA mount media (Invitrogen, Carlsbad CA). Immunohistochemistry of LANA in KS tumors was performed essentially as described previously [55]. Signal amplification was performed by using a peroxidase ABC kit (Vector Laboratories) and followed by diaminobenzidine (DAB; DakoCytomation) reaction.

The images were captured with a Zeiss Axioplan 2 microscope equipped with Zeiss Plan-Neofluar x63/0.75NA objective (Carl Zeiss, Oberkochen, Germany). Images were acquired with a Zeiss Axiocam HRc CCD camera, using Zeiss AxioVision 4.6 SP1 software and processed with Adobe Photoshop 8.0 software (Adobe, San Jose, CA).

For complete list of Materials and Methods see Text S1.

## Supporting Information

Text S1 Nucleophosmin phosphorylation by v-cyclin-CDK6 controls KSHV latency.
(0.05 MB DOC)

Figure S1 NPM is phosphorylated in a v-cyclin dependent manner. (A) Total cell extract from BC-3 cells was separated using gel filtration chromatography, and the fractions marked 1–9 (corresponding approximately to 180–30 kDa) are indicated above the panel. The fractions were resolved by SDS-PAGE (12%) and analyzed by Western blotting with antibodies against NPM, CDK6, and v-cyclin. (B) One half of the peak fractions for NPM were immunoprecipitated with anti-v-cyclin antibodies. Immunocomplexes were resolved by SDS-PAGE and immunoblotted with indicated antibodies. (C) The other half of the peak fractions from panel A were subjected to an in vitro kinase assay towards co-precipitated endogenous proteins followed by separation in 12% SDS-PAGE and autoradiography. Phosphorylated band at 37 kDa (^32^P-37 kDa) as well as phosphorylated p27KIP1 (^32^P-p27KIP1) and p21CIP1 (^32^P-p21CIP1) serving as internal controls for the specificity of the kinase assay are shown. (D) U2OS cells were transiently transfected with Myc-tagged v-cyclin (Myc-v-cyclin), empty vector (Myc), and the indicated NPM expression constructs for wt (eGFP-NPM) and phosphorylation site mutants eGFP-NPM T4A and eGFP-NPM T3A, and analyzed by Western blotting with phospho-NPM antibody (eGFP-pNPM Thr199), anti-GFP antibodies against ectopically expressed NPM (eGFP-NPM), and Myc to confirm expression of Myc-v-cyclin. Tubulin was used as a marker for loading.
(0.31 MB TIF)

Figure S2 NPM is phosphorylated on Thr199 in uninfected endothelial cells. Western blot analysis of whole cell extracts of uninfected and rKSHV.219-infected SLK and EA.hy926 cells. The immunoblots were probed with antibodies against pNPMThr199 and total NPM. Tubulin served as a loading control. Prolonged exposure (5 min) of the Western blot revealed phosphorylated NPM also in the absence of KSHV infection.
(0.17 MB TIF)

Figure S3 NPM phosphorylation is dependednt on v-cyclin, but not on v-FLIP. (A) rKSHV.219-infected SLK cells were transduced with retroviruses expressing control sh-RNA (sh-Scr) or sh-RNA against v-cyclin (sh-v-cyclin). After 48 hours the shRNA expressing cells were transduced either with the control retrovirus (vFLIP -) or retrovirus expressing v-FLIP (vFLIP +). Total RNA was assayed for the abundance of v-cyclin and v-FLIP transcripts at day two following the second retroviral infection. (B) Whole-cell extracts of cells in A were analyzed by immunoblotting with antibodies against pNPMThr199, total NPM, v-cyclin, cyclin A and tubulin. Arrowheads indicate the position of v-cyclin bands.
(0.22 MB TIF)

Figure S4 CDK6 silencing suppresses v-cyclin associated kinase activity. Whole-cell extracts of BC-3 cells expressing control sh-Scr or sh-CDK6 were immunoprecipitated with anti-v-cyclin antibody and assayed for kinase activity toward GST-Rb and Histone H1. Kinase activity was determined by SDS-PAGE (12%) and autoradiography.
(0.14 MB TIF)

Figure S5 Cell cycle analysis of PEL cells upon silencing of CDKs. BC-3 cells expressing sh-Scr, sh-CDK6 or sh-CDK4 were stained with propidium iodide (PI), and their cell cycle profile was determined by measuring total DNA content. Cells were gated for G1, S and G2/M phases of the cell cycle according to the genomic DNA content as determined by PI fluorescence. The percentage of cells in each phase of the cell cycle is indicated.
(0.09 MB TIF)

Figure S6 NPM interacts with LANA in PEL cells. (A) BC-3 cell extract was separated using gel filtration chromatography and the fractions marked 1–8 (corresponding approximately to 700-180 kDa) are indicated above the panel. The fractions were resolved by 10% SDS-PAGE and immunoblotted with antibodies against LANA and NPM. (B) Indicated fractions (marked 1–5) were subjected to reciprocal immunoprecipitations using anti-NPM or anti-LANA antibodies, separated by SDS-PAGE and analyzed for co-precipitated proteins by immunoblotting with the indicated antibodies.
(0.11 MB TIF)

Figure S7 NPM and LANA elution profiles in the control and CDK6-silenced PEL cells. Whole cell extracts of BC-3 cells stably expressing control sh-Scr (top panel) or sh-CDK6 (bottom panel) were separated using gel filtration chromatography. The fractions (marked 0–10) were resolved by 10% SDS-PAGE and immunoblotted with antibodies against NPM and LANA. The peak fractions for NPM and LANA (asterisks) were used for immunoprecipitation with anti-LANA antibody shown in Figure 2B.
(0.39 MB TIF)

Figure S8 Nucleolar localization of fibrillarin in NPM-silenced PEL cells. BC-3 cells transduced with an empty lentiviral vector (mock) or vectors expressing sh-RNAs for NPM (sh1-NPM, sh2-NPM) were stained with an antibody against fibrillarin and the nuclei were visualized by Hoechst staining. All images are representative of multiple fields. Scale bar, 10 µm.
(0.46 MB TIF)

Figure S9 Inhibition of viral reactivation does not rescue LANA-NPM dissociation upon HDAC inhibition. (A) BCBL-1 cells were transfected with siRNA specific for Pim-1 (Pim-1 siRNA), or with control siRNA (Scr siRNA), and subjected 48 hours after transfection for treatment with 1 mM NaB (NaB +) or vehicle (NaB -) for 24 hours. Whole cell extracts were analyzed by Western blotting with antibodies as indicated (left panel) or immunoprecipitated with anti-LANA antibodies (right panel). Protein complexes were resolved by SDS-PAGE and followed by immunoblotting with anti-NPM and -LANA antibodies. Arrowheads indicate the position of possibly acetylated LANA. (B) Total RNAs from the BCBL-1 cells from panel A were assayed for the relative levels of ORF50, ORF57 and K8.1 mRNA by qRT-PCR and normalized to those of human β-actin mRNA. Results were normalized to the values of untreated cells. (C) Whole cell extracts of BCBL-1 cells from A were immunoblotted with a marker for lytic reactivation vIL-6. Tubulin served as a loading control.
(0.41 MB TIF)
