# Supplementary material for: Nucleophosmin Phosphorylation by v-Cyclin-CDK6 Controls KSHV Latency
Source: PLoS Pathog. 2010 Mar 19;6(3):e1000818. doi: 10.1371/journal.ppat.1000818 (PMC2841626; doi:10.1371/journal.ppat.1000818)
Supplement: Text S1 — Nucleophosmin phosphorylation by v-cyclin-CDK6 controls KSHV latency. (0.05 MB DOC) [file ppat.1000818.s001.doc]

# Supplemental Information

**Nucleophosmin phosphorylation by v-cyclin-CDK6
controls KSHV latency**

Grzegorz Sarek^1^, Annika Järviluoma^1^, Henna M. Moore^2^, Sari Tojkander^2^, Salla Vartia^1^, Peter Biberfeld^4^, Marikki Laiho^2,3*^, and Päivi M. Ojala^1,5*§^

^1^Genome-Scale Biology Program, Biomedicum Helsinki & Institute of Biomedicine, ^2^Molecular Cancer Biology Program, Biomedicum Helsinki & Haartman Institute; University of Helsinki, Finland; ^3^Department of Radiation Oncology and Molecular Radiation Sciences, The Johns Hopkins University School of Medicine, Baltimore, Maryland, United States of America; ^4^Department of Pathology and Oncology, Karolinska Institute/Hospital, Stockholm, Sweden, ^5^ Foundation for the Finnish Cancer Institute, Helsinki, Finland

* These authors made equal contributions to the manuscript

^§^Correspondence: [Paivi.Ojala@helsinki.fi](mailto:Paivi.Ojala@helsinki.fi)

**Supplemental Materials and Methods**

## Antibodies

The following primary antibodies against the indicated proteins were used: NPM (32-5200; Zymed); phospho-NPM-Thr199 (3441; Cell Signaling Technology); actin (C-2), CDK4 (H-22), CDK6 (C-21), Cyclin A (C-19), p300 (C-20), Pim-1 (12H8) all from Santa Cruz Biotechnology Inc., and HDAC1 (06-720, Upstate); LANA, and vIL-6 both from ABI Biotechnologies; acetyl-lysine (06-933; Upstate); LANA2/vIRF3 (CM-A807; Novus Biologicals); tubulin (5H1; BD Biosciences Pharmingen), Myc (9E10; Babco, Berkeley, CA), fibrillarin (Abcam; ab5821); GFP (TP401; Torrey Pines Biolabs, Inc., NJ); ORF59 and LANA (gifts from B. Chandran; Rosalind Franklin University of Medicine and Science, IL), and v-cyclin [1]. Quantitative analysis was performed using the ImageJ software package, version 1.42q (National Institutes of Health, USA).

## Plasmids

Mammalian expression vector pcDNA-Myc-v-cyclin was a gift from S. Mittnacht (Institute of Cancer Research, UK). The v-FLIP-pBMN was subcloned from the pAHC-vFLIP [2] and provided by J. Furuhjelm (University of Helsinki). The GFP-NPM expression constructs for the wt pEGFP-C1-B23.1, and phosphosite mutants pEGFP-C1-B23.1-T214/234/237A (T3A) and pEGFP-C1-B23.1-T199/214/234/237 (T4A) were kind gifts from K. Nagata (University of Tsukuba, Japan). The lentiviral expression constructs sh1-NPM and sh2-NPM in pLKO.1 vector backbone were purchased from Open Biosystems (Huntsville, AL). Sh-RNA sequences for NPM were (5’-CCGGGCCAAGAATGTGTTGTCCAAACTCGAGTTTGGACAACACATTCTTGGCTTTTTG-3’) in sh1-NPM (5’-CCGGGCGCCAGTGAAGAAATCTATACTCGAGTATAGATTTCTTCACTGGCGCTTTTTG-3’) in sh2-NPM and (5’- CCTAA GGTTA AGTCG CCCTC GCTCT AGCGA GGGCG ACTTA ACCTT AGG-3’) in non-target control sh-Scr (a gift from J. Klefström, University of Helsinki). Oligonucleotides encoding
sh-RNA targeting v-cyclin (5’-GATCCCCGTTACTGGGCACATGGATGTTCAAGAGACATCCATGTGCCCAGTAACTTTTTGGAAA-3’) or a noncoding, random sequence (5’- GATCCCCGTCACTGGGCATATGGATGTTCAAGAGACATCCATATGCCCAGTGACTTTTTGGAAA-3’) (Promega Corp., Madison, WI) were ligated to pSUPER retroviral vector (a gift from R. Medema, University Medical Center Utrecht). The lentiviral vector pDSL_hpUGIH for the CDK-silencing lentiviruses [3] was obtained from Cell Signaling Technology, Inc. (Danvers, MA). The lentiviral expression plasmids pLenti6/V5-DEST-GFP and pLenti6/V5-DEST-LANA were a gift from J. Haas (University of Edinburgh, UK & Max von Pettenkofer Institut). The double Flag-tagged v-cyclin cDNA in the pBMN retrovirus (KpBMN) was a gift from E. Verschuren (Standford University, Stanford, CA).

## Protein analysis and kinase assay

Immunoblotting, immunoprecipitations, kinase assay and size exclusion chromatography were carried out as detailed [1]. Immunoprecipitation assays shown in figures 2C and 5B were performed by using the Catch and Release v2.0 Immunoprecipitation System (Millipore, Temecula, CA) following the manufacturer's instructions. For immuno-blotting of phosphorylated NPM cells were washed with phosphate-buffered saline (PBS) and lysed in NET buffer (50 mM Tris-HCl [pH 7.5], 0.2% Ipegal, 1 mM EDTA, 150 mM NaCl) in the presence of phosphatase inhibitor cocktail tablets (PhosphoSTOP, Roche), 1mM Na_3_VO_4_, 25 mM beta-glycerophosphate, 1 mM DTT; 2 µg/mL leupeptin; 2 µg/mL pepstatin; and 1.5 µg/mL aprotinin. Proteins were separated in SDS polyacrylamide gels and transferred to nitrocellulose. Blots were blocked with Tris-buffered saline (TBS) containing 5% skim milk for 2 hr and then incubated overnight (4°C) in TBS containing p-NPM (Thr199) antibody (1:1000 dilution). Due to the high abundancy of total NPM in PEL cells, total NPM detection with the anti-NPM antibody (NPM (32-5200; Zymed) was performed with very short exposure times.

## Transfections and RNA interference

U2OS cells were transiently transfected using Fugene 6 transfection reagent (Roche Diagnostics, Indianapolis, IN) according to the manufacturer's instructions. siRNA transfections in BCBL-1 cells were performed essentially as described previously [4].

## Indirect immunofluorescence

BC-3 and BCBL-1 cells were washed twice in PBS, and fixed in 4% paraformaldehyde (PFA) for 15 minutes at room temperature. Cells were washed twice with 3% FCS in PBS and permeabilized with 0.5% NP-40 (Sigma; St. Louis, MO) for 5 minutes at room temperature. Cells were applied onto glass slides and allowed to dry before storage at
–20°C. Adherent cells on coverslips were fixed with 4% (wt/vol) paraformaldehyde (PFA) and permeabilized with 0.1% Triton X-100 for 5 minutes. Immunofluorescence labeling was performed as described previously [3]. The fluorochromes were visualized with a Zeiss Axioplan 2 fluorescent microscope equipped with Zeiss Plan-Neofluar x10/0.3NA, x40/0.5NA and x63oil/1.25NA objectives (Carl Zeiss, Oberkochen, Germany). Images were acquired with a Zeiss Axiocam HRc CCD camera, using Zeiss AxioVision 4.5 SP1 software and processed with Adobe Photoshop 7.0 software (Adobe, San Jose, CA). Quantitative analysis was performed using the ImageJ software package, version 1.42q (National Institutes of Health, USA).

**Cell cycle analysis**

The **cell cycle** profile was determined by **propidium iodide** (PI) staining and flow cytometry **analysis**. Briefly, 2 × 10^6^ **cells** were fixed in ice-cold 70% ethanol. After fixation, the cells were washed in PBS and stained with 30 μg/ml PI (Invitrogen) in PBS supplemented with 50 μg/ml RNase (Sigma-Aldrich) for 1 hour at 37°C. PI stained cells were acquired using a BD-LSR Flow Cytometer (BD Biosciences), and the cell populations were analyzed by CellQuest software (version 3.3; BD).

**Supplemental References**

1. Sarek G, Jarviluoma A, Ojala PM (2006) KSHV viral cyclin inactivates p27KIP1 through Ser10 and Thr187 phosphorylation in proliferating primary effusion lymphomas. Blood 107: 725-732.

2. Ojala PM, Tiainen M, Salven P, Veikkola T, Castanos-Velez E, et al. (1999) Kaposi's sarcoma-associated herpesvirus-encoded v-cyclin triggers apoptosis in cells with high levels of cyclin-dependent kinase 6. Cancer Res 59: 4984-4989.

3. Koopal S, Furuhjelm JH, Jarviluoma A, Jaamaa S, Pyakurel P, et al. (2007) Viral oncogene-induced DNA damage response is activated in Kaposi sarcoma tumorigenesis. PLoS Pathog 3: 1348-1360.

4. Cheng F, Weidner-Glunde M, Varjosalo M, Rainio EM, Lehtonen A, et al. (2009) KSHV reactivation from latency requires Pim-1 and Pim-3 kinases to inactivate the latency-associated nuclear antigen LANA. PLoS Pathog 5: e1000324.
