# Supplementary figures and images for: Nucleophosmin Phosphorylation by v-Cyclin-CDK6 Controls KSHV Latency
Source: PLoS Pathog. 2010 Mar 19;6(3):e1000818. doi: 10.1371/journal.ppat.1000818 (PMC2841626; doi:10.1371/journal.ppat.1000818)

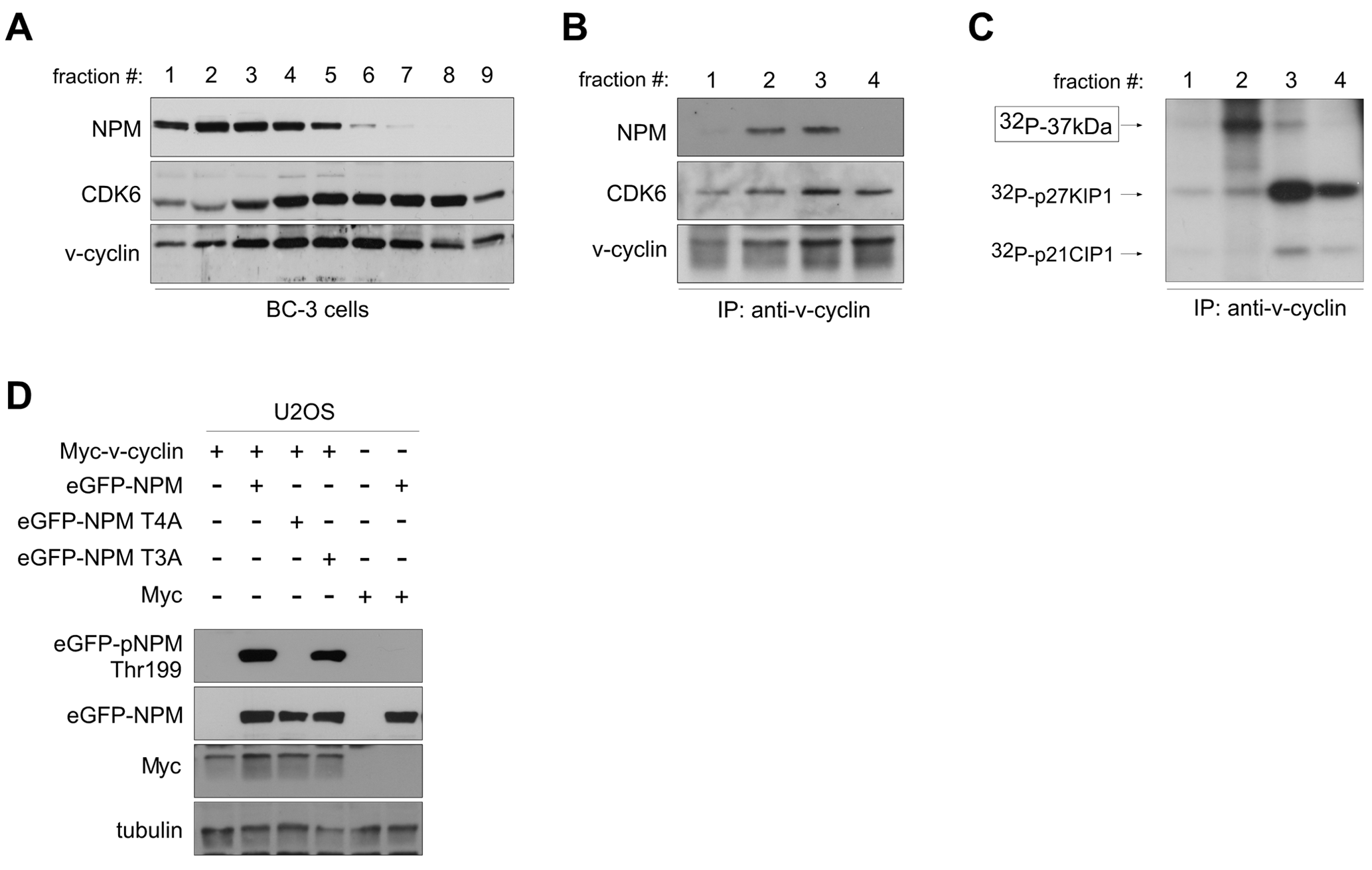

Supplement: Figure S1 — NPM is phosphorylated in a v-cyclin dependent manner. (A) Total cell extract from BC-3 cells was separated using gel filtration chromatography, and the fractions marked 1–9 (corresponding approximately to 180–30 kDa) are indicated above the panel. The fractions were resolved by SDS-PAGE (12%) and analyzed by Western blotting with antibodies against NPM, CDK6, and v-cyclin. (B) One half of the peak fractions for NPM were immunoprecipitated with anti-v-cyclin antibodies. Immunocomplexes were resolved by SDS-PAGE and immunoblotted with indicated antibodies. (C) The other half of the peak fractions from panel A were subjected to an in vitro kinase assay towards co-precipitated endogenous proteins followed by separation in 12% SDS-PAGE and autoradiography. Phosphorylated band at 37 kDa (32P-37 kDa) as well as phosphorylated p27KIP1 (32P-p27KIP1) and p21CIP1 (32P-p21CIP1) serving as internal controls for the specificity of the kinase assay are shown. (D) U2OS cells were transiently transfected with Myc-tagged v-cyclin (Myc-v-cyclin), empty vector (Myc), and the indicated NPM expression constructs for wt (eGFP-NPM) and phosphorylation site mutants eGFP-NPM T4A and eGFP-NPM T3A, and analyzed by Western blotting with phospho-NPM antibody (eGFP-pNPM Thr199), anti-GFP antibodies against ectopically expressed NPM (eGFP-NPM), and Myc to confirm expression of Myc-v-cyclin. Tubulin was used as a marker for loading. (0.31 MB TIF) [file ppat.1000818.s002.tif]

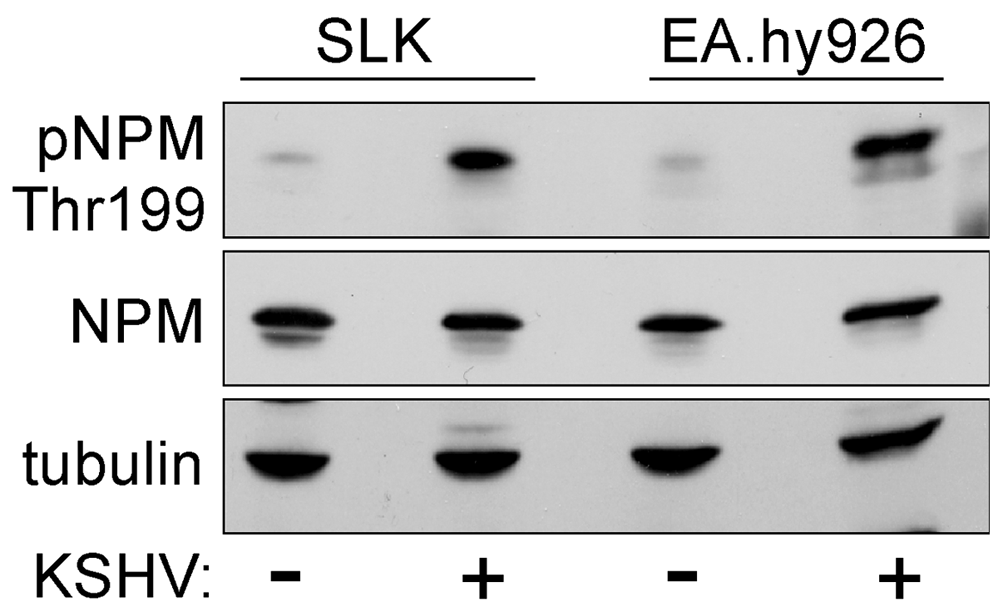

Supplement: Figure S2 — NPM is phosphorylated on Thr199 in uninfected endothelial cells. Western blot analysis of whole cell extracts of uninfected and rKSHV.219-infected SLK and EA.hy926 cells. The immunoblots were probed with antibodies against pNPMThr199 and total NPM. Tubulin served as a loading control. Prolonged exposure (5 min) of the Western blot revealed phosphorylated NPM also in the absence of KSHV infection. (0.17 MB TIF) [file ppat.1000818.s003.tif]

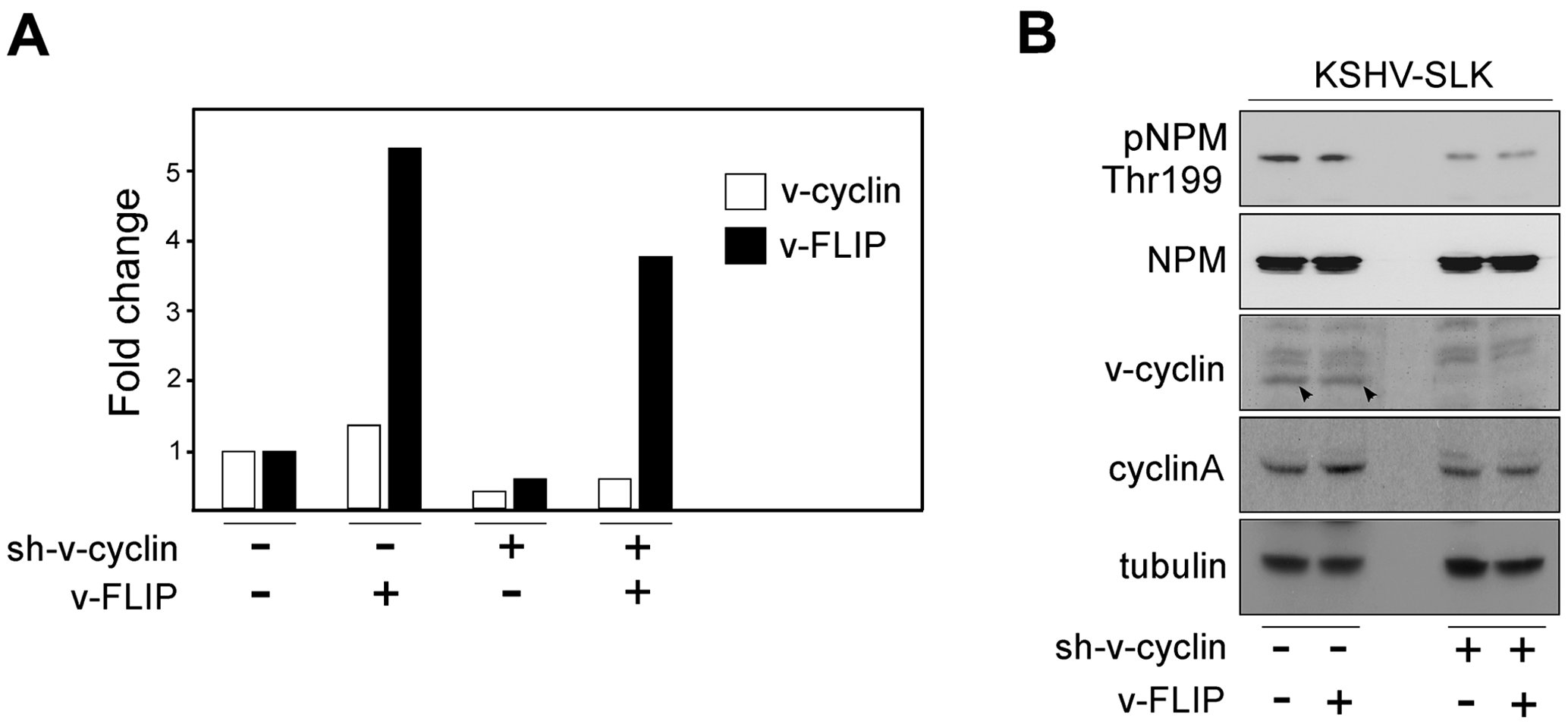

Supplement: Figure S3 — NPM phosphorylation is dependednt on v-cyclin, but not on v-FLIP. (A) rKSHV.219-infected SLK cells were transduced with retroviruses expressing control sh-RNA (sh-Scr) or sh-RNA against v-cyclin (sh-v-cyclin). After 48 hours the shRNA expressing cells were transduced either with the control retrovirus (vFLIP -) or retrovirus expressing v-FLIP (vFLIP +). Total RNA was assayed for the abundance of v-cyclin and v-FLIP transcripts at day two following the second retroviral infection. (B) Whole-cell extracts of cells in A were analyzed by immunoblotting with antibodies against pNPMThr199, total NPM, v-cyclin, cyclin A and tubulin. Arrowheads indicate the position of v-cyclin bands. (0.22 MB TIF) [file ppat.1000818.s004.tif]

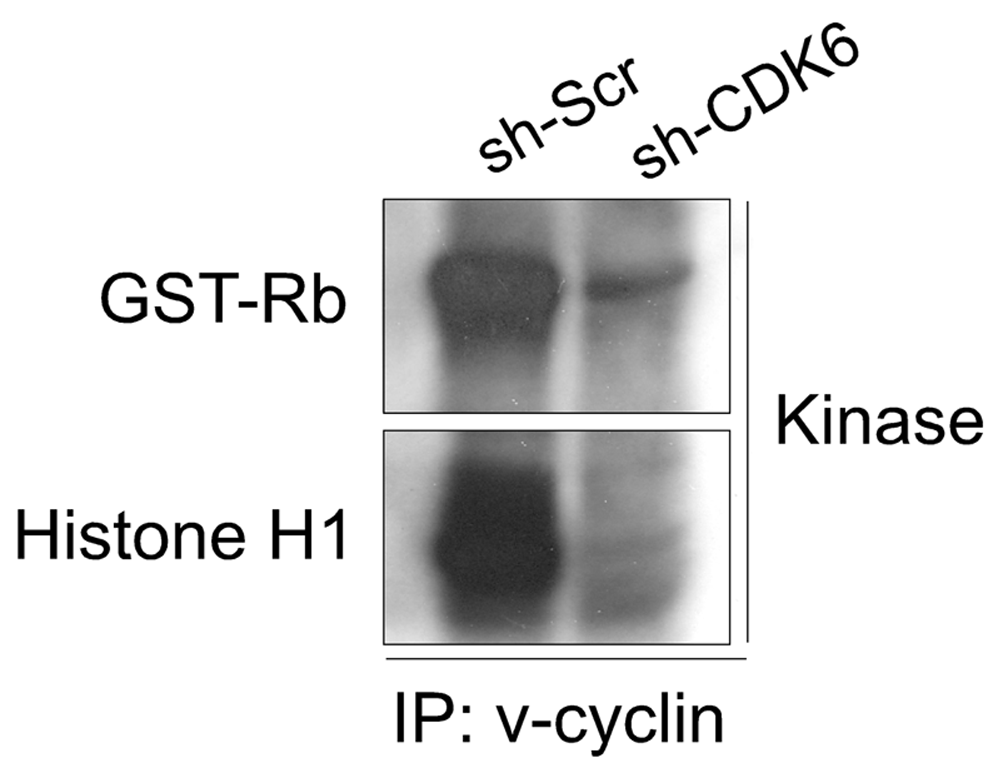

Supplement: Figure S4 — CDK6 silencing suppresses v-cyclin associated kinase activity. Whole-cell extracts of BC-3 cells expressing control sh-Scr or sh-CDK6 were immunoprecipitated with anti-v-cyclin antibody and assayed for kinase activity toward GST-Rb and Histone H1. Kinase activity was determined by SDS-PAGE (12%) and autoradiography. (0.14 MB TIF) [file ppat.1000818.s005.tif]

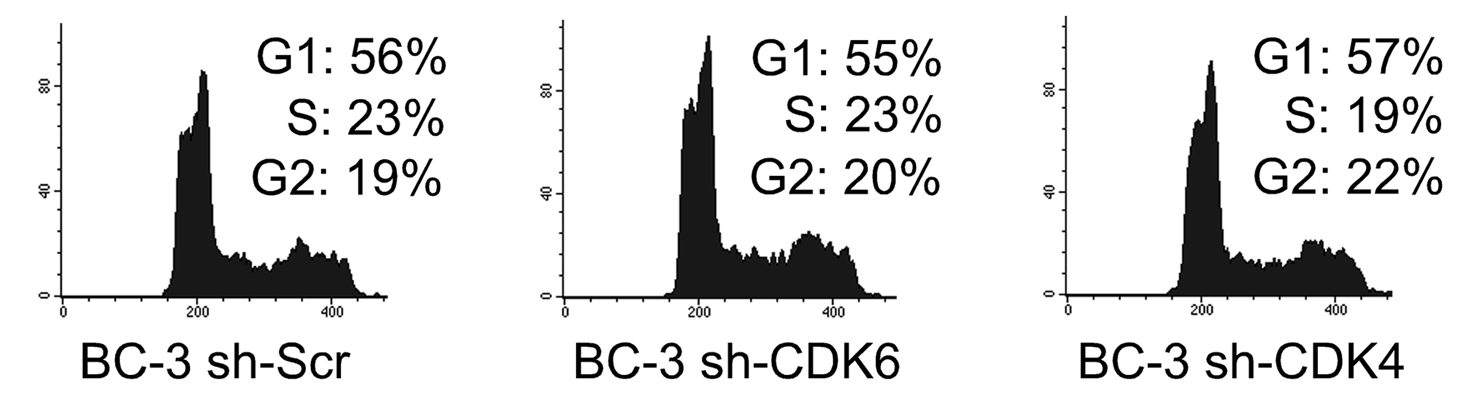

Supplement: Figure S5 — Cell cycle analysis of PEL cells upon silencing of CDKs. BC-3 cells expressing sh-Scr, sh-CDK6 or sh-CDK4 were stained with propidium iodide (PI), and their cell cycle profile was determined by measuring total DNA content. Cells were gated for G1, S and G2/M phases of the cell cycle according to the genomic DNA content as determined by PI fluorescence. The percentage of cells in each phase of the cell cycle is indicated. (0.09 MB TIF) [file ppat.1000818.s006.tif]

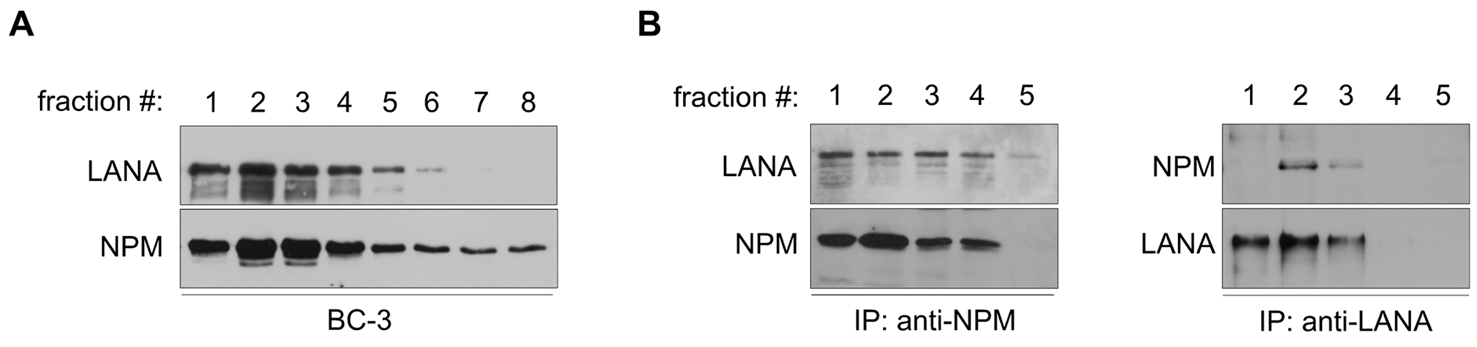

Supplement: Figure S6 — NPM interacts with LANA in PEL cells. (A) BC-3 cell extract was separated using gel filtration chromatography and the fractions marked 1–8 (corresponding approximately to 700-180 kDa) are indicated above the panel. The fractions were resolved by 10% SDS-PAGE and immunoblotted with antibodies against LANA and NPM. (B) Indicated fractions (marked 1–5) were subjected to reciprocal immunoprecipitations using anti-NPM or anti-LANA antibodies, separated by SDS-PAGE and analyzed for co-precipitated proteins by immunoblotting with the indicated antibodies. (0.11 MB TIF) [file ppat.1000818.s007.tif]

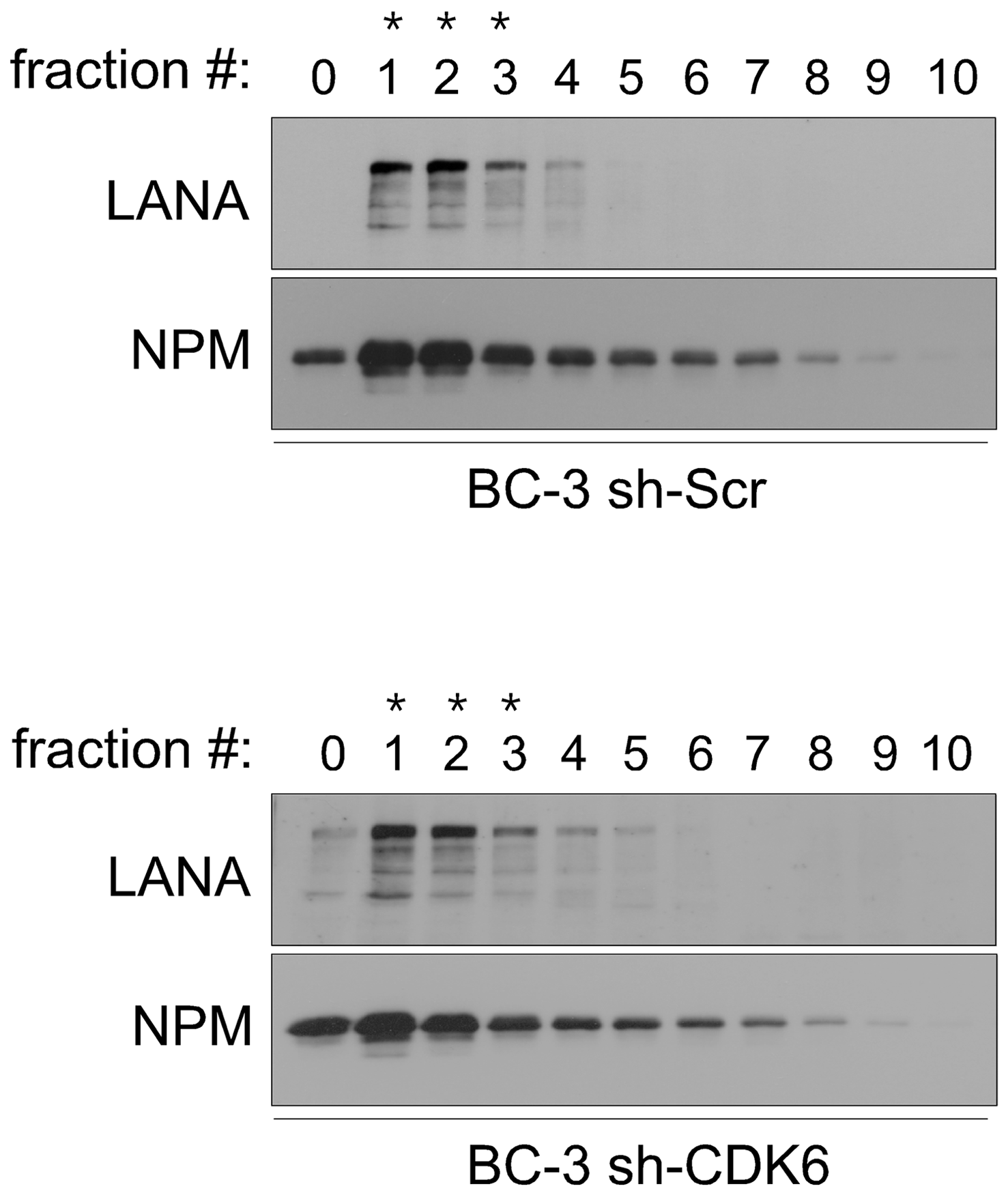

Supplement: Figure S7 — NPM and LANA elution profiles in the control and CDK6-silenced PEL cells. Whole cell extracts of BC-3 cells stably expressing control sh-Scr (top panel) or sh-CDK6 (bottom panel) were separated using gel filtration chromatography. The fractions (marked 0–10) were resolved by 10% SDS-PAGE and immunoblotted with antibodies against NPM and LANA. The peak fractions for NPM and LANA (asterisks) were used for immunoprecipitation with anti-LANA antibody shown in Figure 2B. (0.39 MB TIF) [file ppat.1000818.s008.tif]

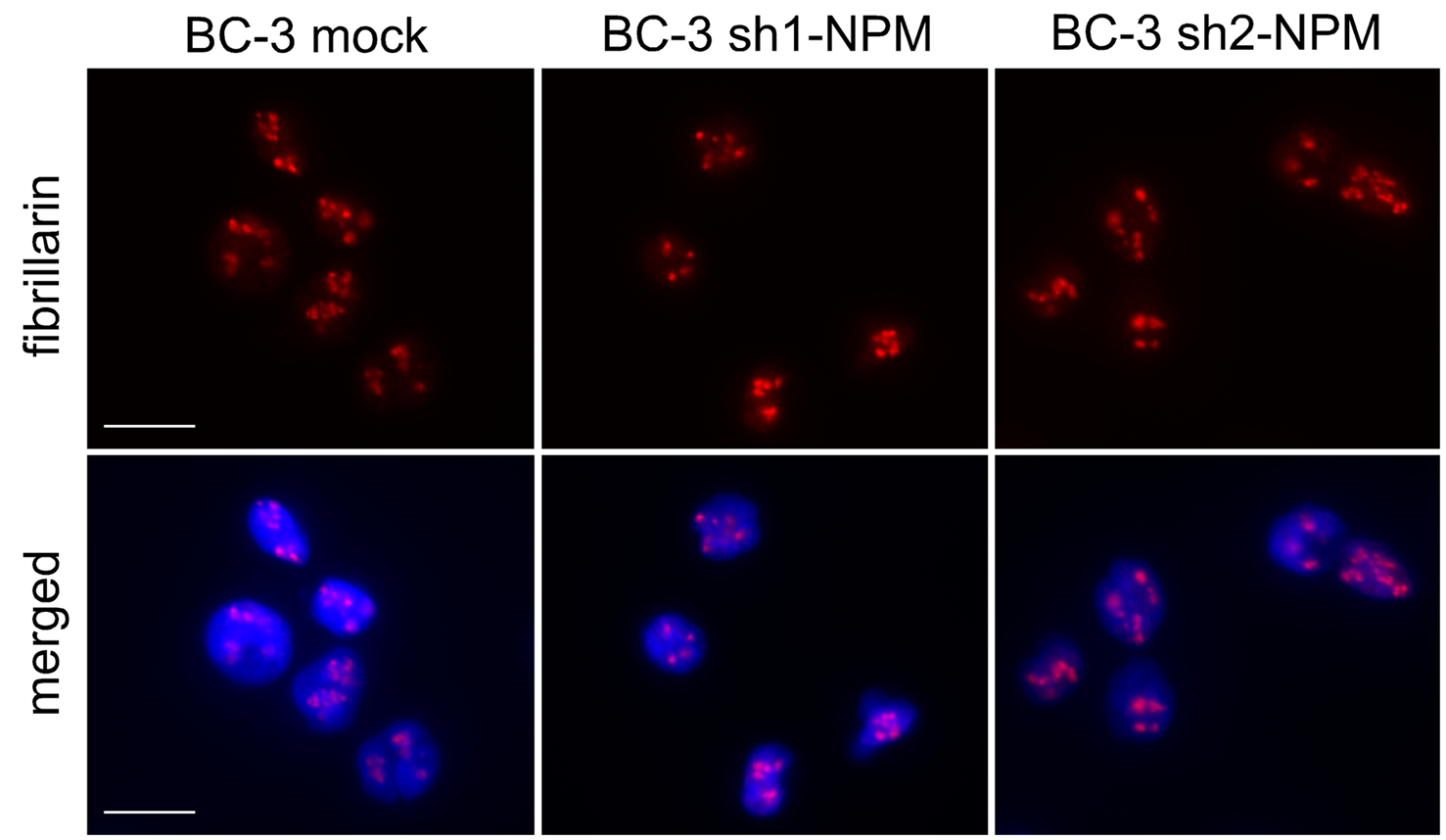

Supplement: Figure S8 — Nucleolar localization of fibrillarin in NPM-silenced PEL cells. BC-3 cells transduced with an empty lentiviral vector (mock) or vectors expressing sh-RNAs for NPM (sh1-NPM, sh2-NPM) were stained with an antibody against fibrillarin and the nuclei were visualized by Hoechst staining. All images are representative of multiple fields. Scale bar, 10 µm. (0.46 MB TIF) [file ppat.1000818.s009.tif]

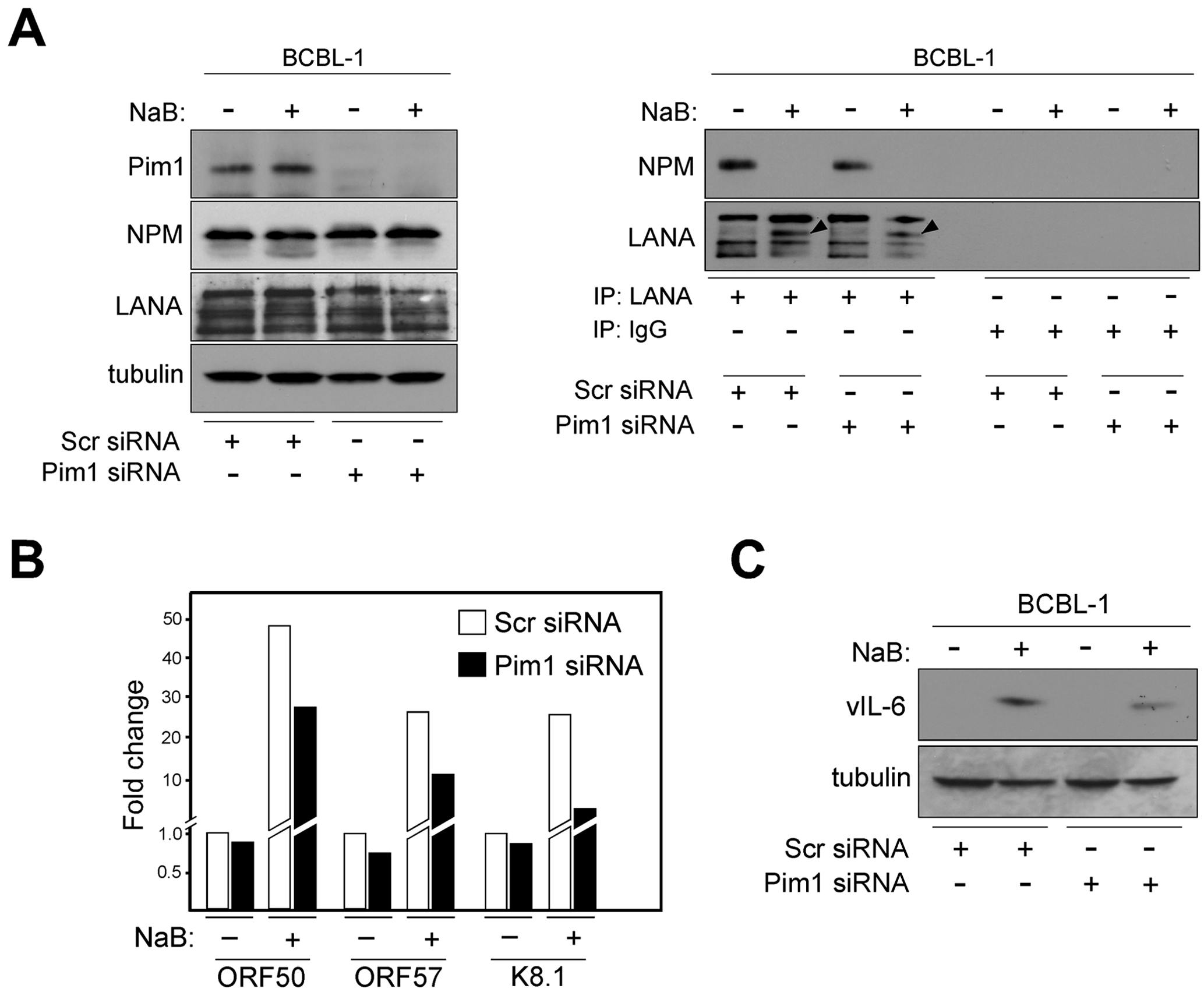

Supplement: Figure S9 — Inhibition of viral reactivation does not rescue LANA-NPM dissociation upon HDAC inhibition. (A) BCBL-1 cells were transfected with siRNA specific for Pim-1 (Pim-1 siRNA), or with control siRNA (Scr siRNA), and subjected 48 hours after transfection for treatment with 1 mM NaB (NaB +) or vehicle (NaB -) for 24 hours. Whole cell extracts were analyzed by Western blotting with antibodies as indicated (left panel) or immunoprecipitated with anti-LANA antibodies (right panel). Protein complexes were resolved by SDS-PAGE and followed by immunoblotting with anti-NPM and -LANA antibodies. Arrowheads indicate the position of possibly acetylated LANA. (B) Total RNAs from the BCBL-1 cells from panel A were assayed for the relative levels of ORF50, ORF57 and K8.1 mRNA by qRT-PCR and normalized to those of human β-actin mRNA. Results were normalized to the values of untreated cells. (C) Whole cell extracts of BCBL-1 cells from A were immunoblotted with a marker for lytic reactivation vIL-6. Tubulin served as a loading control. (0.41 MB TIF) [file ppat.1000818.s010.tif]
